# The role of probiotics in children with autism spectrum disorders: A study protocol for a randomised controlled trial

**DOI:** 10.1371/journal.pone.0263109

**Published:** 2022-02-24

**Authors:** Lingling Zhang, Yiran Xu, Hongwei Li, Bingbing Li, Guiqin Duan, Changlian Zhu

**Affiliations:** 1 Henan Key Laboratory of Child Brain Injury and Henan Clinical Research Center for Child Neurological Disorders, Institute of Neuroscience and The Third Affiliated Hospital of Zhengzhou University, Zhengzhou, China; 2 Center for Child Behavioral Development, The Third Affiliated Hospital of Zhengzhou University, Zhengzhou, China; 3 Center for Brain Repair and Rehabilitation, Institute of Neuroscience and Physiology, University of Gothenburg, Göteborg, Sweden; 4 Department of Women’s and Children’s Health, Karolinska Institutet, Stockholm, Sweden; Fondazione Toscana Gabriele Monasterio, ITALY

## Abstract

**Background:**

Autism spectrum disorder (ASD) is a neurological and developmental condition that begins in infancy or earlier and lasts through the individual’s lifetime. The aetiology and mechanisms of ASD are not yet fully understood, and current treatment comprises mainly education and rehabilitation, without significant improvement in the core symptoms. Recent studies suggest that microbiota change in children with ASD after the ingestion of probiotics may improve the balance of microbiota and thus ASD symptoms.

**Objective:**

The objectives of this study are to evaluate the efficacy of probiotics on the symptoms of children with ASD and the possible mechanisms involved.

**Methods:**

This is a prospective controlled trial. A total of 160 children with ASD will be stratified and allocated to placebo and probiotics groups randomised according to the severity of their ASD symptoms. The probiotics group will be given probiotics supplements orally twice a day for 3 months and the control group will be given a placebo at the same amount, in addition to the baseline therapy of education and rehabilitation. All the children will be evaluated systematically by using different scales, questionnaires before, during, and after 3 months’ treatment, as well as 3 months after discontinuation. The potential impact of probiotics on immunity and inflammation, metabolism, and metagenome will also be investigated.

**Discussion:**

Our previous study showed that the abundance of intestinal flora was greatly different in children with ASD, and that *Bifidobacterium* was associated with the severity of ASD. In the present study, we will investigate the impact of probiotics supplementation on the symptoms of Children with ASD, with the purpose of evaluating the possible therapeutic effects of additives on ASD and of providing a reference for clinical treatment. The results will help to disclose as yet unknown relationship between probiotics and ASD.

**Trial registration:**

This study has been registered with Chinese Clinical Trial Registry (ChiCTR-2000037941).

## Introduction

Autism spectrum disorder (ASD) is one of the most prevalent neurodevelopmental conditions. It is marked by social and communication impairment as well as limited interests and stereotypical behaviours [1]. The incidence of ASD has increased rapidly over time, from 0.31% in 2000 to 1.57% in 2009 [2, 3]. However, the prevalence of ASD varies in different countries; for example, it is currently estimated to affect 1% of the general population in Spain [4] and 1.69% in the United States [5]. A recent study in China showed that the prevalence of ASD there is similar to the estimate numbers in Western countries, that is, approximately 1% [6]. It is estimated that 62.2 million individuals globally live with ASD [7]. A longitudinal study has suggested that people with ASD have poor outcomes, and that the lifetime cost of supporting an individual is a heavy burden [8]. The significant rise of ASD is believed to be attributable to changes in the diagnostic criteria of ASD and increased awareness. Despite several extensive studies, the mechanisms and aetiology of ASD are not yet fully understood. Current studies suggest that various factors are associated with its development; these include genetic, environmental, epigenetic, and microbiome variables and their interactions [9].

Autistic spectrum disorder diagnosis depends mainly on the description of children’s abnormal behaviours by parents and clinical evaluations using different scales, such as the autism diagnosis and observation scale [10]; the autism diagnosis interview scale [11]; the Childhood Autism Rating Scale (CARS) [12]; and the fifth edition of the Diagnostic and Statistical Manual of Mental Disorders (DSM-5) [1]. The main symptoms of ASD can be summarised as the “five no” behaviours [13], namely: no (less) look; no (less) reply; no (less) refers to; no (few) words; and inappropriate words or behaviour. The core symptoms are narrow interests and rigid behaviours. These characteristics seriously affect social interaction, study, and work. Some people with the condition cannot live independently, which places a burden on families and on society in general. There are no widely accepted effective methods to prevent or treat these core symptoms. Currently, treatment of ASD is based mainly on special education, such as applied behaviour analysis (ABA) [14], treatment and education of autistic and related communication handicapped children (TEACCH) [15], relationship development intervention [16], floor time [17], the early start Denver model [18], reciprocal imitation training [19], the preschool autism communication trial [20], and pivotal response training [21]. All these methods are used widely; however, the results show that they do not improve the core symptoms fundamentally or permanently [22].

A range of significant concurrent symptoms, such as gastrointestinal problems [23], sleep problems, anxiety, hyperactivity, and attention deficit hyperactivity disorder [24], are often neglected in targeted interventions. In fact, gastrointestinal and sleep issues are prevalent in Children with ASD [25, 26]. Gastrointestinal symptoms, such as constipation, diarrhoea, abdominal bloating, pain on evacuation, and vomiting have been frequently reported [27]. Research has shown that gastrointestinal symptoms are correlated with various maladaptive behaviours, such as self-injury, aggressive behaviours, restricted stereotypical behaviours, hyperactivity, and language regression [27–29]. However, findings on the relationship between gastrointestinal symptoms and the severity of ASD symptoms are inconsistent [30]. This could be related to differences in methodological approaches to data collection; there are no standardised definitions of gastrointestinal symptoms, and a validated instrument to ascertain gastrointestinal symptoms in individuals with ASD is lacking [23, 27].

Sleep disturbances are often an issue amongst Children with ASD. Sleep is closely related to memory, learning, mood, behaviour, immune response, metabolism, and many other physiological functions [31], and it plays a vital role in the maturation and development of the brain [32]. The most common sleep problems are insomnia, increased bedtime resistance, sleep disordered breathing, early morning wakening, and daytime sleepiness [33]. A number of studies have reported that sleep problems in Children with ASD are associated with behavioural symptoms and their severity [34–36]. Loss of sleep can lead to the accumulation of reactive oxygen species (ROS) and even death [37]. Such problems, along with the core symptoms of ASD, should be taken into account in any treatment.

Gut microbiota have attracted a good deal of attention in recent years. They maintain a stable symbiotic relationship with human beings in the intestines, which are regarded as a “second brain” because of the number of genes encoded by intestinal microbes (150 times the total genes of human cells) [38]. There has been a growing realisation that the gut-brain axis and its regulation by microbiota may play a key role in the biological and physiological basis of neurodevelopmental, age-related, and neurodegenerative disorders [39–41]. Studies have shown that intestinal flora communicates with the brain through the nervous system [42], the immune system [43–46], and the endocrine system [47, 48], which leads to shifts in cognition, social behaviour, and emotion [49, 50]. The abundance of the intestinal flora of Children with ASD undergoes great changes, whether between species or within species. It has been shown that the strains *Akkermansia*, *Coprococcus* and *Ruminococcus* are elevated in Children with ASD [51], and that *Akkermansia* increases regulatory T-cells by suppressing experimental autoimmune encephalomyelitis [52]. *Bacterial lipopolysaccharide* can induce an acute immune response by increasing serum IL-17A levels and promoting ASD-like behaviour in the offspring of mice [53, 54]. However, *Lactobacillus* and *Bifidobacterium* strains have been proven to have anti-inflammatory properties by reducing the level of inflammatory cytokines, such as IL-2, interferon-γ, IL-4, IL-13 and IL-17A, and by increasing the level of anti-inflammatory cytokine IL-10 [55, 56]. These cytokines have been noticed to change in the serum of Children with ASD [57–59].

Studies have revealed that *Collinsella* and *clostridium* are at higher levels in children with ASD [60, 61], and that these strains can produce short-chain fatty acids such as propionic acid and butyric acid. These have neurotoxic effects after passing through the blood-brain barrier, and cause autism-like symptoms (e.g., compulsive interests, abnormal motor movements, and atypical social interactions) in rats [62, 63]. However, probiotics can alleviate the neuroinflammation effectively induced by propionic acid in mice, such as IL-1β and IL-8, and reduce cell apoptosis [64]. Other studies found that probiotics can restore not only the biochemical parameters related to neurotransmission, but also balance the energy metabolism and oxidative stress which are associated with autism [65–67]. Researchers have also observed that some people with ASD may have abnormally porous blood-brain and intestinal barriers, so toxins produced by certain bacteria may enter the blood and reach the brain [48]. Children with autism have been reported to have lower levels of *Bifidobacteria* and higher levels of *Lactobacillus* [68–70]. Gastrointestinal symptoms and quality of life in those with autism were improved by adding *Lactobacillus* and *Bifidobacterium* mixture to the diet [71, 72]. Intestinal microbiota may alleviate constipation through the regulation of gastrointestinal motility or the osmotic effect of metabolites and fermentation products [73], but the precise mechanisms are not clear. Long-term research results have also shown abnormal level of neurotransmitters such as serotonin, dopamine, gamma-aminobutyric acid (GABA), indole, and melatonin [74–75]. Interestingly, 5-hydroxytryptamine (5-HT) levels in the blood are associated with lower gastrointestinal symptoms in ASD [76]. Furthermore, the main neurotransmitters and hormones of GABA and melatonin are involved in sleep promotion, while serotonin, glutamate, and acetylcholine are mainly responsible for wakefulness [77]. Gamma-aminobutyric acid (GABA) is one of the major inhibitory neurotransmitters from the decarboxylation of glutamate at glutamate decarboxylase. Decreased N-methyl-D-aspartate receptor function on GABAergic neurons can lead to postsynaptic glutamate neuron disinhibition, excessive glutamate release, and neurotoxicity [78]. Disturbed GABA levels are commonly found in autistic children or rodents with neurological damage, which may be a key factor in the pathogenesis of autism [79–81]. The production of those neurotransmitters and hormones may be effected by intestinal flora such as *Streptococcus thermophilus* and indigenous spore forming bacteria (Sp) *Lachnoclostridium bolteae*,*Lachnoclostridium hathewayi*, and *Flavonifractor plautii* [69, 82]. With the deepening understanding of probiotics, research on emerging microbial strains has led to vaccines with systemic immunomodulatory activity [83] and revealed the production of neuroactive metabolites [84] and inhibitory activity against infectious microorganisms in the intestines [85]. In recent years, studies of probiotics in Children with ASD have shown great potential benefits in terms of the amelioration of gastrointestinal dysfunction [86], malnutrition, and the severity of ASD symptoms [72, 87–89]. As for non-pharmacological and relatively risk-free, probiotics may be an ideal option for ASD.

Above all, it has been shown that the intestinal flora can adjust the level of intestinal metabolites such as neurotransmitters and hormones through a change in diversity. This could have an impact on behaviour, interest, mood, the gastrointestinal tract, and sleep patterns in children. However, the precise schema and pathways are not yet fully understood. Therefore, we hypothesise that probiotic supplementation may help improve abnormal gut metabolites and immunity in Children with ASD, thus improving ASD symptoms.

## Methods

### Study design and participants

**Fig 1:**
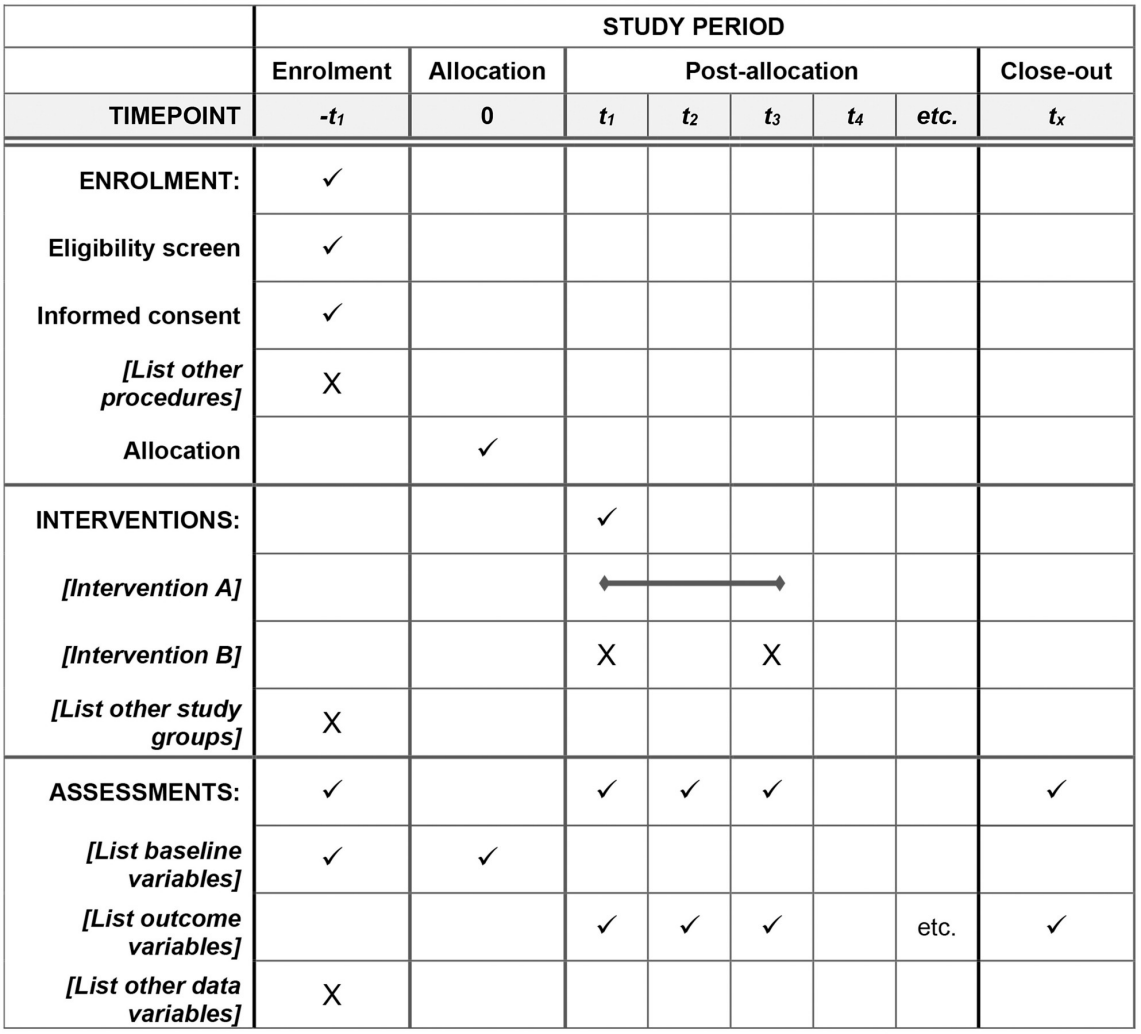
The schedule of enrolment, interventions, and assessments. This list mainly includes three time points, which are the start time of intervention T0, the end time of the supplement T1 and the end time of washout period T2.

We will carry out a randomised, double-blind, placebo-controlled, parallel group trial to evaluate the efficacy and safety, lyophilized powder mixtures (S1 Table), contenting *Lactobacillus*, *Bifidobacteria* and *Streptococcus thermophilus*, to be taken 10 billion colony units twice a day for 3 months, followed by a 3-month washout period and then a 6-month follow up, as add-on therapy in children with ASD irrespective of any ABA and TEACCH maintenance therapy. The placebo group will be given maltodextrin. At each time point, all participants will receive the following assessments and investigations of Autism Treatment Evaluation Checklist (ATEC), CARS, social responsiveness scale second edition, children’s sleep health questionnaire, survey of dietary patterns, gastrointestinal assessment questionnaires and the Bristol stool chart. The children will be required to avoid taking antibiotics during this period. If they do they will have to be excluded (Fig 1). Patients aged 3 to 12 years will be eligible if they are defined as having ASD by two child psychologists using DSM-5 [1], but if they meet one of the following exclusion criteria they will not be allowed to take part: (1) they have taken antibiotics or antifungal drugs within three months; (2) they have a special diet, such as a gluten free diet, a casein diet, or a special carbohydrate diet; (3) they have taken prebiotics, probiotics, or antioxidants within the previous three months; (4) they have had diarrhoea, fever, or other clear inflammatory reactions within the past week; (5) they have neurological symptoms such as epilepsy; (6) they have Crohn’s disease, inflammatory bowel disease, food intolerance, or any other gastrointestinal diseases; (7) they have a liver disease; (8) they have type 1 diabetes mellitus; (9) they have a confirmed diagnosis of Rett syndrome or any other genetic metabolic disease. The exit criteria are: (1) children who do not take the probiotics as required; (2) children whose blood and stool samples provide no biological information; and (3) participants cannot complete DSM-5, CARS, and the other compulsory scales for ASD evaluation. All the participants are from the Center for Child Behavioral Development at the Third Affiliated Hospital of Zhengzhou University. This clinical trial has been registered at the Chinese Clinical Trial Registry (ChiCTR2000037941), and approved by the ethical committee of the Third Affiliated Hospital of Zhengzhou University (2020–56). All the participants will join the research on a voluntary basis. An information fact sheet and a consent form will be distributed to the front-line healthcare providers and parents after an introductory talk of the program. They understand the benefits and risks involved in the program, and their participation in the program is voluntary. They have the right to question any part of the procedure and can withdraw at any time without negative consequences. The completed consent form will be collected by the research team before the study commences.

### Randomisation and masking

**Fig 2:**
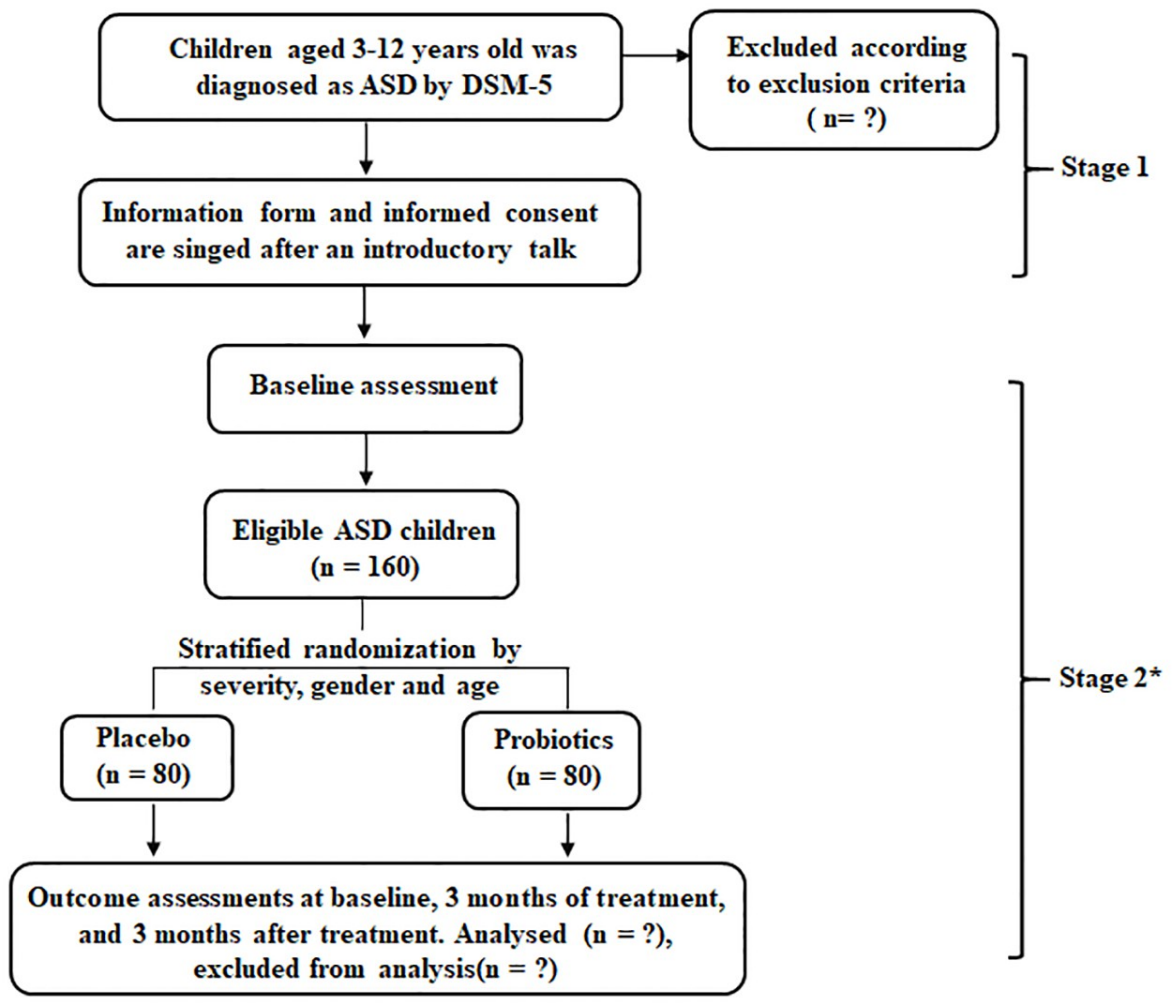
Schematic diagram of study design. The assessment mainly includes the severity of autism, gastrointestinal problems, sleep assessment, parental stress assessment, eating habits and diet structure. The experimental results mainly include the results of the metabolism of blood, urine and stool, and the changes in the intestinal flora, as well as the changes in immune cells and cytokines; Entire experiment includes 3-month medication and 3-month elimination. *Adverse effects are monitored throughout the period.

Randomisation was stratified according to severity, gender, age (3–6 years or 7–12 years). The ratio of male to female in the study will be about 5:1 based on the registration of in patient ASD. We will use block randomisation within sites (Fig 2). Participants will be allocated (1:1) to the probiotics or placebo group using a concealed random allocation from a computer-generated random numbers table produced by Python (a cross-platform computer programming language). The additives used for the placebo group will have the same packaging, taste, and weight as the experimental group, except for the ingredients. All participants and doctors will be blind to the allocation of treatments.

### Sample size calculation

The sample size will be calculated using Epitools (https://epitools.ausvet.com.au/onemean). The expected difference between the probiotic and placebo groups is based on a previous study on ASD, in which the standard deviation value of behaviour score was 8.32 in the ASD group [89]. To detect a clinically significant difference in the outcome measures with the condition of 90% power (α = 0.05; two-sided), 67 children are required for each group. Based on clinical study experience [90], we presume a drop-out rate of 19% through infection, efficacy, compliance, use of antibiotics, or other unforeseeable factors, implying a minimum of 80 children for each group.

### Procedures

Before the trial, all the children will be evaluated carefully, and the research topic will be explained to the parents of the Children with ASD who meet the study’s selection criteria. If they are then willing to participate in the trial, they will receive the supplements free of charge for three months and carry out the relevant inspections and tests. They will sign an informed consent agreement. They will be able to consult with the doctors if they have any questions when they are administering the supplements, but they must follow the requirements of the agreement. If they are not willing to continue the study for some reason, they can withdraw. All the potential side effects will be monitored and all the participants will be recalled at 3 months after intervention for the systematic physical examination and liver as well as kidney function analysis. The potential costs will be covered by indemnity for negligent harm. The participants will be asked to provide a one tube faeces sample 0, 1, 2, and 3 months after taking the supplements, as well as at the end of 3 months of wash out, and a tube of blood with heparin anticoagulation at 0 and 3 months. To guarantee quality all samples must be transported within 12 hours at low temperatures or they will be rejected. Other than the samples used for flow cytometry detection, which has to be completed within 12 hours, all samples will be aliquots and frozen in a freezer at -80°C.

### Quality control

The trial datasets will be housed on the project files of Henan Key Laboratory of Child Brain Injury. All the principal investigators are given to access to the cleaned datasets without identifying participant information. Safety and interim analyses will be done when half of the patients are recruited, and these will be reviewed by the Safety and Data Monitoring Committee. The validity of results from the intervention can only be verified by project manager who have full access to the complete final dataset. To ensure the quality of the sample and the reliability of the data, we will set up a We chat communication group to remind the parents or guardians to carry out the required treatments and examinations regularly. Furthermore, if the children have any doubts or problems during the treatment, they can contact the doctors at any time. We will provide information about probiotics to the group to increase their understanding of the trial; this may help reduce the drop-out rate. All patient information will be confidential and will be managed by a specific researcher.

### Primary outcome measure

#### a) Assessment of core symptoms

All parents/guardians will be asked to complete an ATEC [91] to evaluate changes in core symptoms in Children with ASD at the time of the baseline, the third month, and the sixth month respectively. The scale consists of four subscales: speech, perception, social interaction, and behaviour. The total score is 0–179; the higher the score, the more serious the condition. The reduction rate of ATEC score before (S_1_) and after (S_2_) treatment will be used as the efficacy index (N), N = (S_1_- S_2_)/S1 × 100%. Markedly effective: N_M_ ≥ 50%, Effective: N_E_: 20%–50%, Ineffective: N_I_ < 20%. Total effective rate (N_T_) = (N_M_ + N_E_) / total cases × 100%.

#### b) Assessment of severity

CARS [12] and social responsiveness scale [92] will be used. CARS scale consists of 15 items with a total score of 60 points. A score of less than 30 points means no autism is present, and a score of 30–60 points means autism is present; 30–36 points signifies moderate autism, and 37–60 points with at least five items and a score more than three points signifies severe autism. However, the social responsiveness scale mainly shows the abnormalities in the interactive social behavior of subjects and the difficulties in daily social behavior. This scale has a total of 65 items, which are divided into a 5-point scale. The score is calculated and converted into a total score, which is divided into normal, mild, moderate and moderate.

### Secondary outcome measures

#### a) Assessment of gastrointestinal symptoms

Gastrointestinal symptoms will be assessed by gastrointestinal assessment questionnaires [93] and the Bristol Stool Chart [94]. First, in accordance with the Bristol stool chart, we will classify the stool into seven levels, namely, type I: a dry nut-like ball-shaped stool, which is difficult to discharge; type II: sausage-like but very hard; type III: sausage-like surface cracks; type IV: sausage-like or snake-like, smooth and soft; type V: soft lump; clear-cut edge (and easy to discharge); type VI: loose fragments, broken edges, or mushy stool; type VII: watery stool, with no solid parts. Type IV is an ideal healthy state. We will use the questionnaires scale to conduct a further survey of the gastrointestinal condition of the participants. The scale is composed of 15 items. Each item uses a four-level method of 0–3 points, The symptoms for the corresponding scores are: (0) asymptomatic, indicating that the patient does not have this feeling; (1) mild, i.e., occasional symptoms; (2) moderate, i.e., frequent symptoms; and (3) severe, i.e., persistent symptoms.

#### b) Assessment of sleep

The Children’s Sleep Habits Questionnaire is based on the International Classification of Sleep Disorders [95], which is used for children aged 4–12 years. The scale is filled in by the parents, who monitor their child’s sleep patterns over the previous four weeks and choose a typical week. Each item uses a 3-level score based on the frequency of sleep-related behaviours, from low to high from 1 to 3 points, respectively. There is a total of 52 items; the higher the number, the greater the children’s sleep disorders. The questionnaire comprises eight common sleep disorders in children, namely: 1) sleep impedance; 2) delay in falling asleep; 3) sleep duration; 4) sleep anxiety; 5) waking at night; 6) parasomnia; 7) sleep breathing disorders; and 8) daytime sleepiness. The questionnaire uses the total score > 41 points as the standard for evaluating sleep disorders, and defines the standard for sleep disorders as a frequency of more than two nights a week in each item [35].

#### c) Assessment of parenting stress

The Parenting Stress Index-Short Form [96] contains 36 items with three dimensions: parental distress, parent-child dysfunction, and the difficult child. Each dimension contains 12 items. The scale uses a 5-point scale, from 1 to 5 respectively: *strongly disagree*, *disagree*, *unsure*, *agree*, and *strongly agree*. The total score can range from 36 to 180 points. The higher the score, the greater the pressure. The parenting distress dimension reflects the parents’ abilities in raising the children, handling conflicts with their spouse, social support, and life pressures. The personal interaction disorder dimension evaluates the parents’ attitude towards the child’s failure to meet expectations and the child’s inability to interact well. The child’s difficulty dimension is to investigate parents’ reactions to children’s emotions, resistance, and non-cooperation.

#### d) Assessment of eating habits

The Children’s Eating Behaviour Questionnaire [97] is a valid and reliable 35-item measure of children’s eating behaviours. It has eight subscales: food-responsiveness, food fussiness, emotional over-eating, enjoyment of food, desire to drink, satiety-responsiveness, slowness in eating, and emotional under-eating. Higher scores reflect more extreme eating behaviours.

#### e) The survey of dietary patterns

The structure of food has a great impact on the gastrointestinal tract. The scale used concerns the consumption of staple foods, vegetables, fruits, eggs, meat, water, and antibiotics, and related sleep and exercise conditions (S2 Table). The "OKOK" mobile phone health management software is used to record the daily diet.

#### f) Metabolomics of faeces and blood

Metabolic profiling of samples will be performed on an Agilent 1290 Infinity LC system (Agilent Technologies, Santa-Clara, California, USA) coupled with an AB SCIEX Triple TOF 6600 System (AB SCIEX, Framingham, MA, USA). Chromatographic separation will be implemented on ACQUITY UPLC BEH Amide 1.7 μm (2.1 × 100 mm) columns for both positive and negative models. The column temperature will be set at 25°C.

#### g) Changes in inflammatory profiles

Inflammatory profiles will be detected by flow cytometry. All FACES processes will be completed within 12 hours by using the antibodies of surface and intracellular markers of peripheral blood mononuclear cells (PBMC). PBMCs will be isolated by density gradient centrifugation as described previously [98]. Flow cytometry will be used to detect cytokines IL-17A, IL-10, IFN-γ, IL-6, IL-21, IL-2, IL-1β, IL-8 produced by CD4^+^ and CD8^+^ cells and IL-1β, IL-10, IL-6, IL-8 produced by monocytes. Briefly, PBMCs will be stimulated with PMA/ionomycin for 4 h in the presence of BD GolgiStop, as previously described [99]. Then PBMCs will be washed and stained for CD4^+^ and CD8^+^ cells and monocytes. Cytokine staining was performed with fixed permeability method for anti-IL-17A, anti-IL-10, anti-IFN-γ, anti-IL-6, anti-IL-21, anti-IL-2, anti-IL-1β, anti-IL-8. Samples will be taken and analysed by BD FACSCelesta flow cytometer and Flowjo software.

#### h) Diversity and richness of flora

A total of 1μg DNA per sample was used as input material for the DNA sample preparations. Sequencing libraries were generated using NEBNext^®^ Ultra^™^ DNA Library Prep Kit for Illumina (NEB, USA) following the manufacturer’s recommendations, and index codes will be added to attribute sequences to each sample. The DNA sample will be fragmented by sonication to a size of 350bp, then DNA fragments will be end-polished, A-tailed, and ligated with the full-length adaptor for Illumina sequencing with further PCR amplification. Finally, PCR products will be purified (AMPure XP system) and libraries will be analysed for size distribution by Agilent2100 Bioanalyzer and quantified using real-time PCR. The clustering of the index-coded samples will be performed on a cBot Cluster Generation System in accordance with the manufacturer’s instructions. After cluster generation, the library preparations will be sequenced on an Illumina HiSeq platform and paired-end reads generated.

### Statistical analyses and power

The severity and ASD before and after administration of probiotics will be analysed in accordance with the requirements of the scale previously described [66]. Relevant questionnaires will be analysed by Chi-square test. Statistical significance will be set at *p* < 0.05. All raw data can be available at the Website (http://beta.fairsharing.org/3752) after we finish the study.

The entire study has multiple omics measurements that are taken at different points. The metagenome sequencing data will be analysed using meta-seq metagenome pipeline. The specific process is as follows. First, the raw reads will be extracted from the sequencing results, and fast quality control (version 0.11.9) with default parameters will be used to evaluate their quality. Adaptor sequences in the reads will be trimmed using Trimmo matic (Version 0.38). Braken2 (version 2.0.8b), Kraken, and Kaiju (version 1.6.3) will be used for taxonomic classification of the metagenome short reads. The meta-seq pipeline will involve the use of meta SPAdes (version 3.13.2) and MEGAHIT (version 1.2.9) for short read assembly. Meta Prodigal (version: 2.6.3) will be employed for gene prediction of the assembled metagenomic contigs. The abundance of each ORF (measured with TPM) will be quantified with Salmon (version 1.1.0). The functional information of the ORFs will be described with KEGG, AMR and COG. R package DESseq2 will detect the differential abundance ontology or taxonomy catalogue. Assembled contigs with more than 1500bp will be used to reconstruct the metagenome-assembled Genome using Meta BAT (version 2.12.1). CheckM (version 1.0.7) will enable evaluation of the quality and completeness of the reconstructed draft genome. GTDB-tk (version 1.0.2) will be used to identify the lineage classification of the obtained metagenome-assembled Genome.

The raw UPLC-Q-TOF/MS data will be converted to mzXML files using ProteoWizard msConverte tool and then processed using XCMS online software. The heat plot of metabolites will be obtained through Multi Experiment Viewer software 4.9.0 after unit variance scaling for each metabolite. The online KEGG database (http://www.genome.jp/kegg/) will be used for the identification of metabolic pathways.

## Discussion

The prevalence of ASD continues to increase worldwide, but valid therapeutic strategies to improve core symptoms are lacking. This places additional demands on affected families and on society as a whole. Therefore, there is an urgent need to develop such strategies. The present study aims to explore whether probiotics supplementation has the potential to improve the symptoms of ASD and the possible mechanisms by which they may work.

Our previous study showed that the diversity of gut microbiota in Children with ASD changed significantly, and the changes in *Bifidobacterium* was correlated with the severity of ASD [60]. This suggests that the gut is associated with the occurrence and development of ASD, so probiotics may have potential value in clinical applications. It has been reported that gut microbiota from ASD donors have been transplanted to sterile mice that exhibit autistic behaviour. When the ASD mice were treated with candidate microbial metabolites, their abnormal behaviour improved significantly, and the excitability of brain neurons were regulated [100]. A Systematic review found that altered gut microbiota in autistic children may trigger intestinal leakage, of which short-chain fatty acids, lipopolysaccharide, calprotectin, lysozyme and lysozyme can be used as biomarkers for early detection of intestinal leakage [101]. This indicates that gut microbiota could be a target for ASD treatment.

Probiotics supplements can protect the intestinal barrier and reduce effectively the occurrence of intestinal diseases [102, 103]. Oral Bif195 can reduce the risk of small intestinal disease caused by acetylsalicylic acid [104]. In addition, probiotics have certain effects on the immune system. Nasal administration of *Rhamnose* GG prevents the development of birch pollen-induced allergic asthma in a strain-specific manner [105]. Furthermore, in the current consensus, the role of probiotics is not considered to be solely mediated by microflora; in fact, their metabolic function may go beyond their effect on colonial microflora, and this may open the door to a wider range of probiotic possibilities. A meta-analysis found that polyunsaturated fatty acids (especially eicosapentaenoic acid) can assist in the treatment of depression and improve attention deficit/hyperactivity disorder [106].

Previous studies have shown that probiotics or their metabolites regulate changes in immune cells, cytokines, and emotional behaviour, and have great potential therapeutic effect. Therefore, it is necessary to carried out more comprehensive studies on the pathogenesis of autism to find further effective treatment methods. This protocol describes a multi-level, multi-faceted, independent, and closely related randomised controlled study. All the training, assessment, and testing will be completed by specially trained and experienced child psychologists or rehabilitation trainers and nurses. They will carry out the training according to the requirements of the agreement. In the meanwhile, all parents or guardians of the children will receive professional training to learn about the relevant precautions before they participate in the trial. The specific information collected will be recorded in detail, and those who do not meet the requirements will be excluded. Given the influence of subjective factors, the questionnaire is not only scored by parents, but also evaluated by the doctors who participate in the intervention or related tests.

In sum, the object of this study is to clarify whether probiotics mixing is an effective treatment for children with ASD in daily clinical practice. Meanwhile, it will also help us to clarify the changes in the immune and intestinal systems of the condition, laying a foundation for discovering its pathogenesis. In addition, probiotics are much more risk-free than drugs; some ingredients are common in infant dairy products. Therefore, the Children with ASD or parents are likely to be more willing to accept them and to cooperate in the research.

## Supporting information

S1 Checklist SPIRIT 2013 checklist: Recommended items to address in a clinical trial protocol and related documents*. (DOC)

S1 Table List of probiotic mixtures. (DOCX)

S2 Table Eating habits questionnaire. (DOCX)

S1 File (DOCX)

S2 File (DOCX)

## Abbreviations

ABA: applied behavior analysis
ASD: autism spectrum disorder
ATEC: autism treatment evaluation checklist
CARS: childhood autism rating scale
DSM-5: diagnostic and statistical manual of mental disorder-5
PBMC: peripheral blood mononuclear cells
TEACCH: treatment and education of autistic and related communication handicapped
