# Supplementary material for: The role of probiotics in children with autism spectrum disorders: A study protocol for a randomised controlled trial
Source: PLoS One. 2022 Feb 24;17(2):e0263109. doi: 10.1371/journal.pone.0263109 (PMC8870536; doi:10.1371/journal.pone.0263109)
Supplement: S1 Table — (DOCX) [file pone.0263109.s002.docx]

**Supplementary Table 1. List of probiotic mixtures**

| **Strains** | **Dosage (Billion Colony Forming Unit)** |
| --- | --- |
| *Lactobacillus reuteri* TR02 | 0.6 |
| *Lactobacillus Swiss* TR11 | 0.4 |
| *Lactobacillus plantarum* TR22 | 0.4 |
| *Lactobacillus casei* TR01 | 0.6 |
| *Lactobacillus rhamnosus* TR08 | 0.6 |
| *Lactobifidobacterium* TR101 | 0.6 |
| *Bifidobacterium brevis* TR103 | 0.4 |
| *Bifidobacterium longum* TR17 | 0.2 |
| *Animal bifidobacterium* TR20 | 0.6 |
| *Lactobacillus giannis* TR520 | 0.2 |
| *Streptococcus thermophilus* TR14 | 0.2 |
| *Streptococcus acidophilus* TR03 | 0.2 |
