# Supplementary material for: The role of probiotics in children with autism spectrum disorders: A study protocol for a randomised controlled trial
Source: PLoS One. 2022 Feb 24;17(2):e0263109. doi: 10.1371/journal.pone.0263109 (PMC8870536; doi:10.1371/journal.pone.0263109)
Supplement: S2 Table — (DOCX) [file pone.0263109.s003.docx]

**Supplementary table 2. Eating habits questionnaire**

| **Please fill in according to your child's situation in the last month.** | | | | | | | | | | | | | | | | | | | |
| --- | --- | --- | --- | --- | --- | --- | --- | --- | --- | --- | --- | --- | --- | --- | --- | --- | --- | --- | --- |
| 1 | Height: | Weight: | | | | | | | | | | | | | | | | | |
| 2 | Taste of diet (multiple choices available) | 1. Light | 2. Sweet | | | | | | 3. Spicy | | | | 4. Salt addictive | | | | | | |
|  |  | 5. Acid addictive | | | | | | | | | | | | | | | | | |
| 3 | Diet structure | 1. Balanced meat and vegetables | | | | | | | | | | | | | | | | | |
|  |  | 2. Based on meat and vegetables | | | | | | | | | | | | | | | | | |
|  |  | 3. Based on vegetarian food | | | | | | | | | | | 4. Based on vegetarian diet | | | | | | |
|  |  | 5. Based on snacks | | | | | | | | | | | | | | | | | |
| 4 | How many times of eating breakfast on average in a week? | 1.0-1 times | | | | 2.2-3 times | | | | | | 3.4-5 times | | | | | 4. Every day | | |
| 5 | What nutritional health products have you taken? | 1. Nutrition (protein, vitamins from bee products) | | | | | | | | | | | | | | | | | |
|  |  | 2. Fortified (calcium, zinc and other minerals) | | | | | | | | | | | | | | | | | |
|  |  | 3. Functional type (fish oil, lecithin) | | | | | | | | | | | | | | | | | |
|  |  | 4. No supplement | | | | | | | | | | | | | | | | | |
| 6 | How much weight did you gain in the past 12 months? | 1. No increase | | | | 2.1-3kg | | | | | | 3.3-6kg | | | | | 4.7-10kg | | |
| 7 | How much height has increased in the past 12 months? | 1. No increase | | | | 2.1-3cm | | | | | | 3.3-6cm | | | | | 4.7-10cm | | |
| 8 | Antibiotics commonly used in the past month (multiple choices available) | 1. Never used | | | | | | | 2. Chloramphenicol | | | | | | | 3. Lincomycin | | | |
|  |  | 4. β-lactamases: penicillin, ampicillin sodium, amoxicillin, piperacillin, cephalexin, cefadroxil, cefazolin sodium | | | | | | | | | | | | | | | | | |
|  |  | 5. Aminoglycosides: streptomycin, gentamicin, kanamycin, amikacin, small nomicin | | | | | | | | | | | | | | | | | |
|  |  | 6. Tetracyclines: tetracycline, oxytetracycline, doxycycline, minocycline, etc. | | | | | | | | | | | | | | | | | |
| 9 | How many times of eating snacks on average in a week? | 1. No | 2. 100-300g | | | | | | | | | | 3. 200-400 g | | | | | | |
|  |  | 4. 300-500g | | | | | | | | | | | 5. More than 800g | | | | | | |
| 10 | Food structure | 1.Mainly rice, small amount of coarse-grain potatoes | | | | | | | | | | | | | | | | | |
|  |  | 2. Mainly white flour, small amount of coarse-grain potatoes | | | | | | | | | | | | | | | | | |
|  |  | 3. Rice white noodles and coarse grain potatoes are basically the same amount. | | | | | | | | | | | | | | | | | |
|  |  | 4. Coarse grains and potatoes are mainly used, a small amount of rice white noodles | | | | | | | | | | | | | | | | | |
| 11 | How much of staple food do you take daily? | 1. No | 2. 100-300g | | | | | | 3. 200-400 g | | | | 4. 300-500g | | | | | | 5. More than800g |
| 12 | Picky eaters | 1. No | 2. Mild (0-3 kinds of foods are not eaten) | | | | | | | | | | | | | | | | |
|  |  | 3. Moderately picky eaters (3-5 kinds of foods are not eaten) | | | | | | | | | | | | | | | | | |
|  |  | 4. Severely picky eaters (only eat less than 5 kinds of food) | | | | | | | | | | | | | | | | | |
| 13 | How much high-cholesterol foods do you take daily? | 1.Less than 50g | | | | 2.50-100g | | | | | | 3.100-200g | | | | | 4.morethan200g | | |
| 14 | How much red meat do you take daily? | 1.Less than 50g | | | | 2.50-100g | | | | | | 3.100-200g | | | | | 4.morethan200g | | |
| 15 | How much white meat or eggs does do you take daily? | 1.None | | | | 2. 0-100g | | | | | | 3.100-200g | | | | | 4.morethan200g | | |
| 16 | How much fruit do you take daily? | 1. None | | | | 2. 0-100g | | | | | | 3.100-200g | | | | | 4.morethan200g | | |
| 17 | How much vegetables do you take daily? | 1. None | | | | 2. 0-100g | | | | | | 3.100-200g | | | | | 4.morethan200g | | |
| 18 | How much milk products (excluding yogurt) do you take daily? | 1. None | | | | 2.0-240ml | | | | | | 3.240-480ml | | | | | 4.morethan480ml | | |
| 19 | How much yogurt or probiotic beverages are consumed in a week? | 1. Every day | | | | 2. 0-2 times a week | | | | | | | | | 3. 2-3 times a week | | | | |
|  |  | 3. 3-5 times a week | | | | | | | | | | 4. Basically do not drink | | | | | | | |
| 20 | A shelf life for yogurt or milk? | 1.7-21 days without probiotics | | | | | | | | | | 2.7-21 days without probiotics | | | | | | | |
|  |  | 3.6 months (such as: Pure Zhen, Ai Muxi, Moslian) | | | | | | | | | | | | | | | | | |
| 21 | How often do you eat eggs ? | 1. 1 a day | | | | 2. 2 a day | | | | | | 3. 1-3 a week | | | | | 4. Do not eat | | |
| 22 | How often do you eat fermented foods? | 1. Regularly | | | 2. 2-3 times a week | | | | | | | | 3. Rarely | | | | 4. Don't eat | | |
| 23 | The amount of water consumed per day (including beverages) | 1.1500ml or more | | | | | | | | | | 2.1000-1500ml | | | | | | | |
|  |  | 3.500-1000ml | | | | | | | | | | 4.500ml or less | | | | | | | |
| 24 | Do you have a habit of drinking soup or porridge? | 1. Meals and meals | | | | | | | | | | 2. 1-3 times a day | | | | | | | |
|  |  | 3. More than 3 times a week | | | | | | | | | | 4. Less than 3 times a week | | | | | | | |
| 25 | Defecation in the past week | 1. Basically smooth | | | | | | | | | | 2. Habits are not smooth | | | | | | | |
|  |  | 3. Stool formation | | | | | | | | | | 4. Stool formation is difficult | | | | | | | |
| 26 | Duration of sleep disturbance | 1. No | | 2.0-1 hour | | | | | | 3.2-3 hours | | | | 4.3 hours or more | | | | | |
| 27 | Nap duration | 1. Never | | 2.0-30 minutes | | | | | | | 3.30-60 minutes | | | | | | 4.1-2 hours | | |
|  |  | 5. More than 2 hours | | | | | | | | | | | | | | | | | |
| 28 | How long is the baby's static time (except sleep time) daily? | 1. Less than 1 hour | | | | | 2.1-2 hours | | | | | | 3.3-4 hours | | | | | 4.5-6 hours | |
|  |  | 5.7 hours or more | | | | | | | | | | | | | | | | | |
| 29 | How long is the baby's medium-intensity activity every day? | 1. Rarely participate | | | | | | 2.0-1 hours | | | | | 3.1-2 hours | | | | | 4.3-4 hours | |
|  |  | 5.4 hours or more | | | | | | | | | | | | | | | | | |
| 30 | 30 What food does the baby is allergic to |  | | | | | | | | | | | | | | | | | |
