# Supplementary material for: The role of probiotics in children with autism spectrum disorders: A study protocol for a randomised controlled trial
Source: PLoS One. 2022 Feb 24;17(2):e0263109. doi: 10.1371/journal.pone.0263109 (PMC8870536; doi:10.1371/journal.pone.0263109)
Supplement: S1 File — (DOCX) [file pone.0263109.s004.docx]

**Study protocol:**

**The role of probiotics in children with autism spectrum disorders**

Principal Investigator and Organizer:

Changlian Zhu, MD, PhD

Department of Pediatrics

The Third Affiliated Hospital

Zhengzhou University

Zhengzhou 450052, China

Tel: 0086-371-66903050

Email: [zhuc@zzu.edu.cn](mailto:zhuc@zzu.edu.cn)

Center for Brain Repair and Rehabilitation

Institute of Neuroscience and Physiology

University of Gothenburg

Gothenburg 40530, Sweden.

Tel: 0046-31-786 3339

Email: changlian.zhu@neuro.gu.se

Status: Sept.1^st^, 2019

**Study protocol:**

**The role of probiotics in children with autism spectrum disorders**

**Study Group**

A group of child rehabilitation specialist from the Department of Child Development and Behavior of the Third Affiliated Hospital of Zhengzhou University and the Key Laboratory of Pediatric Brain Injuries of Henan Province Zhengzhou, China.

**Principal Investigator:**

Changlian Zhu, MD, PhD. Department of Pediatrics, the Third Affiliated Hospital, Zhengzhou University, Zhengzhou 450052, China. E-mail: zhuc@zzu.edu.cn Tel: +86 371 66903051

**Major scientists involved in the Probiotics Study:**

Lingling Zhang, the Third Affiliated Hospital of Zhengzhou University and Center for Brain Repair and Rehabilitation, Zhengzhou, China.

Yiran Xu, MD, the Third Affiliated Hospital of Zhengzhou University and Center for Brain Repair and Rehabilitation, Zhengzhou, China.

Hongwei Li, PhD student, the Third Affiliated Hospital of Zhengzhou University and Center for Brain Repair and Rehabilitation, Zhengzhou, China.

Bingbing Li, Research assistant, the Third Affiliated Hospital of Zhengzhou University and Center for Brain Repair and Rehabilitation Zhengzhou, China.

Guiqin Duan, MD, Department of Child Development and Behavior, the Third Affiliated Hospital of Zhengzhou University, Zhengzhou, China.

Changlian Zhu, PhD, the Third Affiliated Hospital of Zhengzhou University and Center for Brain Repair and Rehabilitation, Institute of Neuroscience and Physiology, Sahlgrenska Academy, University of Gothenburg, Gothenburg, Sweden.

**Synopsis**

This randomized, controlled study aims to figure out the role of probiotics in core symptoms and severity in children with autism. Secondary objectives are to determine whether probiotics regulate gastrointestinal disorders and sleep disturbances which often troubled children with autism, as well as its effect on blood and fecal metabolites, intestinal flora, and immune system.

Patients aged 3 to 12 years will be eligible if they are defined as having ASD by two child psychologists using DSM-5, but if they meet one of the following exclusion criteria they will not be allowed to take part: (1) they have taken antibiotics or antifungal drugs within three months; (2) they have a special diet, such as a gluten free diet, a casein diet, or a special carbohydrate diet; (3) they have taken prebiotics, probiotics, or antioxidants within the previous three months; (4) they have had diarrhoea, fever, or other clear inflammatory reactions within the past week; (5) they have neurological symptoms such as epilepsy; (6) they have Crohn’s disease, inflammatory bowel disease, food intolerance, or any other gastrointestinal diseases; (7) they have a liver disease; (8) they have type 1 diabetes mellitus; (9) they have a confirmed diagnosis of Rett syndrome or any other genetic metabolic disease.

All participants will be provided written informed consent before they approved to take the probiotics as required. The patients will be divided into treatment group and control group according to their severity, gender and age randomly. The treatment group will receive probiotics treatment for three months before three months withdrawal. The control group will be given the same amount of maltodextrin. The treatment and nursing of the two groups are in accordance with the same standards.

Therapeutic effect evaluation will be done at 1, 2, 3, 6 and 12 months after taking probiotics. 3, 6, 12, and 18 months by the ATEC, CSHQ, Birstol and other scales. The primary outcome was whether probiotics could alleviate poor language and cognition, narrow interest ranges, and gastrointestinal and sleep disorders in children with autism.

**Background**

**Ethical considerations**

Gut microbiota have attracted a good deal of attention in recent years. They maintain a stable symbiotic relationship with human beings in the intestines, which are regarded as a “second brain” because of the number of genes encoded by intestinal microbes (150 times the total genes of human cells) ^[1]^。Studies have shown that intestinal flora communicates with the brain through the nervous system ^[2]^、the immune system ^[3-6]^ and the endocrine system ^[7, 8]^，which leads to shifts in cognition, social behaviour, and emotion ^[9, 10]^。The abundance of the intestinal flora of Children with ASD undergoes great changes, whether between species or within species. Such as *Akkermansia*、*Coprococcus* and *Ruminococcus* are elevated and *Bifidobacterium* are decreased in Children with ASD^[11-14]^。The gastrointestinal symptoms and quality of life in ASD were improved by adding a mixture of *Lactobacillus* and *Bifidobacterium* to the diet ^[15, 16]^。

In this study, each volunteer’s guardian will sign an informed consent before taking probiotics, they be informed of the study process and precautions, and they can withdraw from the study for any reason during the process.

**Epidemiology**

Autism spectrum disorder (ASD) is one of the most prevalent neurodevelopmental conditions. It is marked by social and communication impairment as well as limited interests and stereotypical behaviours ^[17]^. The incidence of ASD has increased rapidly over time, from 0.31% in 2000 to 1.57% in 2009^[18, 19]^.It is estimated that 62.2 million individuals globally live with ASD ^[20]^. Moreover，the prevalence of ASD varies in different countries; for example, it is currently estimated to affect 1% of the general population in Spain^[21]^, 1.69% in the United States^[22]^ and 1% in China^[23]^. The core symptoms of ASD are narrow interests and rigid behaviours. A longitudinal study has suggested that people with ASD have poor outcomes, and that the lifetime cost of supporting an individual is a heavy burden^[24]^。.

**Probiotics**

The definition of probiotics was defined by the International Association for Probiotics and Probiotics Science is "a substrate that is selectively utilized by host microorganisms conferred health benefits" ^[25]^. Common probiotics: (1) Lactobacillus (such as Lactobacillus acidophilus, *Lactobacillus casei*, Lactobacillus Jennings, Lactobacillus Ramanei, etc.); (2) Bifidobacterium (such as *bifidobacterium longum*, *bifidobacterium brevis*, *bifidobacterium ovale*, *bifidobacterium thermophilus*, etc.); (3) Gram-positive cocci (such as *Streptococcus faecalis*, *Lactococcus*, *Streptococcus* intermediae, etc.); (4) Some yeasts and enzymes can also be classified as probiotics.

**Effects**

Probiotics has three main uses: (1) Treatment of ASD;(2) Balance gastrointestinal tract;(3) Regulating disturbed sleep

In recent years, studies on probiotics in children with ASD have shown that probiotics have great potential benefits in improving gastrointestinal dysfunction, malnutrition and ASD symptom severity ^[16, 26-28]^. In addition, its metabolic products also have a vital influence on the development of the nervous system.

For example, 5-hydroxytryptamine (5-HT) levels in the blood are associated with lower gastrointestinal symptoms in ASD^[29]^. Furthermore, the main neurotransmitters and hormones of gamma-aminobutyric acid (GABA) and melatonin are involved in sleep promotion, while serotonin, glutamate, and acetylcholine are mainly responsible for wakefulness^[30]^.

**Adverse effects**

Probiotics can cause uncomfortable gastrointestinal symptoms such as abdominal pain and diarrhea depending on the individual^[31]^.

**Study objectives**

The objectives of this randomized, controlled study are 1) to determine whether the probiotics treatment could relieve the core symptoms, gastrointestinal tract and sleep disorders of ASD. 2) to find out possible early biological markers of ASD. and 3) to figure out the possible pathogenesis of ASD.

**Hypotheses**

Primary: Probiotic supplements could relieve the core symptoms of autism，such as the narrow range of interests or activities and repetitive and stereotypical behavior.

Secondary(a): Probiotics can help balance gastrointestinal disorders in children with autism, such as constipation and diarrhea.

Secondary(b): Metabolites from the gut flora help improve sleep disorders.

Secondary(c): The metabolites of intestinal flora contribute to the improvement of immunity.

**Study design**

Experimental group: Oral probiotic mixture of 5 billion CFU/5g, twice a day in the morning and once in the evening respectively, for 3 months.

Control group: The same amount of maltodextrin, treatment course and usage were the same as experimental group.

Both groups were given the same nursing and diagnosis and treatment plan.

**PATIENT SELECTION**

**Inclusion Criteria**

1. Patients aged 3 to 12 years, gender unlimited;
2. Who diagnosed as ASD by DSM-5[17] definitely;
3. 3. Participate in the study and sign the informed consent voluntarily;

**Exclusion Criteria**

1. they have taken antibiotics or antifungal drugs within three months;
2. they have a special diet, such as a gluten free diet, a casein diet, or a special carbohydrate diet;
3. they have taken prebiotics, probiotics, or antioxidants within the previous three months;
4. they have had diarrhoea, fever, or other clear inflammatory reactions within the past week;
5. they have neurological symptoms such as epilepsy;
6. they have Crohn’s disease, inflammatory bowel disease, food intolerance, or any other gastrointestinal diseases;
7. they have a liver disease;
8. they have type 1 diabetes mellitus;
9. they have a confirmed diagnosis of Rett syndrome or any other genetic metabolic disease.

E**xit criteria**:

1. children who do not take the probiotics as required;
2. children whose blood and stool samples provide no biological information;
3. participants cannot complete DSM-5, CARS, and the other compulsory scales for ASD evaluation.

The children with autism who met the inclusion criteria and the exclusion criteria were randomly grouped according to gender, age and severity.

**STUDY PROCEDURES**

Before the trial, all the children will be evaluated carefully, and the research topic will be explained to the parents of the Children with ASD who meet the study’s selection criteria. If they are then willing to participate in the trial, they will receive the supplements free of charge for three months and carry out the relevant inspections and tests. They will sign an informed consent agreement. They will be able to consult with the doctors if they have any questions when they are administering the supplements, but they must follow the requirements of the agreement. If they are not willing to continue the study for some reason, they can withdraw. All the potential side effects will be monitored and all the participants will be recalled at 3 months after intervention for the systematic physical examination and liver as well as kidney function analysis. The potential costs will be covered by indemnity for negligent harm. The participants will be asked to provide a one tube faeces sample 0, 1, 2, and 3 months after taking the supplements, and a tube of blood with heparin anticoagulation at 0 and 3 months. To guarantee quality all samples must be transported within 12 hours at low temperatures or they will be rejected. Other than the samples used for flow cytometry detection, which has to be completed within 12 hours, all samples will be aliquots and frozen in a freezer at -80℃.

**Consent**

This study has been approved by the Ethics Committee of the Third Affiliated Hospital of Zhengzhou University (2020-56). Randomisation will be done after the researcher explain the details of the clinical study to the guardian of the children and sign the informed consent. Consent written must be documented by a witness.

**Diagnosis and treatment**

**Diagnosis**

It meets the diagnostic criteria of ASD established in the American Diagnostic Statistical Manual of Psychiatry 5th edition^[17]^: (1) persisting deficits in social communication and social interaction in different environments;(2) limited and repetitive interests, behaviors, or activities;(3) These symptoms exist in early development; (4) These symptoms cause significant impairments in professional, social, or other vital functions; (5) Mental retardation or global developmental retardation cannot better explain these symptoms;

Meeting the above criterion can be diagnosed as ASD, of which (1)(2) is the core symptom of ASD. The child was diagnosed with autism spectrum disorder need by three senior specialists in the same field.

**Assessment of** **core symptoms**

All parents/guardians will be asked to complete an Autism Treatment Evaluation Checklist (ATEC) ^[32]^ to evaluate changes in core symptoms in Children with ASD at the time of the baseline, the third month, and the sixth month respectively. The scale consists of four subscales: speech, perception, social interaction, and behaviour. The total score is 0-179; the higher the score, the more serious the condition. The reduction rate of ATEC score before (S1) and after (S2) treatment will be used as the efficacy index (N), N = (S1- S2)/S1 × 100%. Markedly effective: NM ≥ 50%, Effective: NE: 20%−50%, Ineffective: NI < 20%. Total effective rate (NT) = (NM + NE) / total cases × 100%.

**Assessment of severity**

CARS will be used ^[33]^. The scale consists of 15 items with a total score of 60 points. A score of less than 30 points means no autism is present, and a score of 30-60 points means autism is present; 30-37 points signifies moderate autism, and 37-60 points with at least five items and a score more than three points signifies severe autism.

**Assessment of sleep**

The Children’s Sleep Habits Questionnaire (CSHQ) is based on the International Classification of Sleep Disorders^[34]^. Each item uses a 3-level score based on the frequency of sleep-related behaviours, from low to high from 1 to 3 points, respectively. There is a total of 52 items; the higher the number, the greater the children’s sleep disorders. The questionnaire comprises eight common sleep disorders in children, namely: 1) sleep impedance; 2) delay in falling asleep; 3) sleep duration; 4) sleep anxiety; 5) waking at night; 6) parasomnia; 7) sleep breathing disorders; and 8) daytime sleepiness. The questionnaire uses the total score > 41 points as the standard for evaluating sleep disorders, and defines the standard for sleep disorders as a frequency of more than two nights a week in each item^[35]^.

**Assessment of gastrointestinal symptoms**

Gastrointestinal symptoms will be assessed by gastrointestinal assessment questionnaires（GSRS）^[36]^ and the Bristol Stool Chart^[37]^.

**Major Rehabilitation Training**

Rehabilitation training includes applied behavioral analysis therapy (ABA) ^[38]^and structured education (TEACCH) ^[39]^.

**Sample and data collection**

Peripheral Blood: A vacuum vasculture containing heparin anticoagulant was used to collect 1-2mL of peripheral venous blood at the time of baseline, three months after probiotics administration, and the deadline.

Stool samples: Fecal samples were collected at the baseline, three months after probiotics administration, and the deadline, respectively. Temporary storage in -20℃ refrigerator within 1 hour, transported in ice bags within 24 hours, -80℃ storage .

The information of candidate's gender, age, history of asphyxia, family history, history of allergies, diet and the mother's history of pregnancy, hypertension during pregnancy, mode of delivery need to collect. At the same time, the diagnostic results of DSM-5 and the evaluation results of ATEC, CARS, CSHQ, GSRS and so on also need to be recorded. These information can be used for subgroup analysis.

**Follow-up**

**1,2,3, 6,12months:** Assessment of Gastrointestinal tract, sleep, speech and communication skills, social skills, perceptual skills, health and physical behavior according to scale. (blinded assessment by certified study personnel)

**Primary Outcome Measure**

- 1. Efficacy evaluation
  2. Severity of disease

**Secondary outcome measures**

1. Gastrointestinal symptoms
2. Sleep
3. Parental pressure
4. Eating habits
5. Diet pattarns
6. Metabolites in Blood and fecal
7. Immune cells
8. Abundance of intestinal flora

**STATISTICAL ANALYSIS**

**Randomisation and masking**

Randomisation was stratified according to severity, gender, age (3−6 years or 7−12 years). Participants will be allocated (1:1) to the probiotics or placebo group using a concealed random allocation from a computer-generated random numbers table produced by Python (a cross-platform computer programming language). The additives used for the placebo group will have the same packaging, taste, and weight as the experimental group, except for the ingredients. All participants and doctors will be blind to the allocation of treatments.

**Sample size calculation**

The sample size will be calculated using Epitools (<https://epitools.ausvet.com.au/onemean>). The expected difference between the probiotic and placebo groups is based on a previous study on ASD, in which the standard deviation value of behaviour score was 8.32 in the ASD group^[31]^. To detect a clinically significant difference in the outcome measures with the condition of 90% power (α = 0.05; two-sided), 67 children are required for each group. Based on clinical study experience ^[40]^, we presume a drop-out rate of 19% through infection, efficacy, compliance, use of antibiotics, or other unforeseeable factors, implying a minimum of 80 children for each group.

**Data Monitoring and Interim Analysis**

**DATA COLLECTION & MONITORING**

Data collection and monitoring of study centre will be done by the centre co-ordinators. Data will be passed on to the Department of Medical Documentation of the Third Affiliated Hospital of Zhengzhou University, China, for analysis. Safety and interim analyses will be done when half of the patients are recruited, and these will be reviewed by the Safety and Data Monitoring Committee.

Centre co-ordinators will meet every month to maintain the consistency of procedures when documenting site visits, and sample chart reviews by centre co-ordinators will be reviewed by the principal investigator.

**INTERIM ANALYSIS**

Safety analysis will be executed to compare the adverse effects in the probiotics and control groups if there erupt large batch of adverse gastrointestinal reactions or other allergic symptoms within two weeks after taking probiotics.

Interim analysis will be done when 40 of the patients in each group have been recruited and 3 months of data are available.

If the trial is stopped prematurely, the patients already included in the study will be followed up as scheduled by this study protocol.

**Final Analysis**

The final analysis will be based on intent-to-treat and carried out after follow-up is closed for all patients in the study.

The analysis will include

- Source data listing
- An elementary descriptive analysis of all variables that were observed
- Assessment of the data quality that was achieved
- Description of study patients
- Assessment of drop-outs and the comparability of groups
- Evaluation of efficacy, including scheduled subgroups analysis
- Explorative analysis if further interesting results are suspected
- Biometrical assessment of the validity of the study results

In case there is an unexpectedly high proportion of dropouts, the most important parts of the analysis will still be done for all admitted patients (full sample analysis) as well as for those patients that can be used to test the efficacy of the treatment.

**Financial Support**

This study is supported by Henan Province Key Scientific Research Project (171100310200) and Henan Province Medical Research Project (LHGJ20190349).

**LITERATURES**

1. Qin J, Li R, Raes J, Arumugam M, Burgdorf KS, Manichanh C, et al. A human gut microbial gene catalogue established by metagenomic sequencing. Nature. 2010;464(7285):59-65. doi: 10.1038/nature08821. PubMed PMID: 20203603; PubMed Central PMCID: PMCPMC3779803.

2. Han W, Tellez LA, Perkins MH, Perez IO, Qu T, Ferreira J, et al. A Neural Circuit for Gut-Induced Reward. Cell. 2018;175(3):665-78 e23. doi: 10.1016/j.cell.2018.08.049. PubMed PMID: 30245012; PubMed Central PMCID: PMCPMC6195474.

3. Mukherji A, Kobiita A, Ye T, Chambon P. Homeostasis in intestinal epithelium is orchestrated by the circadian clock and microbiota cues transduced by TLRs. Cell. 2013;153(4):812-27. doi: 10.1016/j.cell.2013.04.020. PubMed PMID: 23663780.

4. Erny D, Hrabe de Angelis AL, Jaitin D, Wieghofer P, Staszewski O, David E, et al. Host microbiota constantly control maturation and function of microglia in the CNS. Nat Neurosci. 2015;18(7):965-77. doi: 10.1038/nn.4030. PubMed PMID: 26030851; PubMed Central PMCID: PMCPMC5528863.

5. Lamas B, Richard ML, Leducq V, Pham HP, Michel ML, Da Costa G, et al. CARD9 impacts colitis by altering gut microbiota metabolism of tryptophan into aryl hydrocarbon receptor ligands. Nat Med. 2016;22(6):598-605. doi: 10.1038/nm.4102. PubMed PMID: 27158904; PubMed Central PMCID: PMCPMC5087285.

6. Irene Tsilionia ABP, b, Harry Pantazopoulosc,1, Sabina Berrettac, Pio Contid, Susan E. Leemane,2,, and Theoharis C. Theoharidesa b, f,2. IL-37 is increased in brains of children with autism spectrum disorder and inhibits human microglia stimulated by neurotensin. PNAS. 2019. doi: 10.1073/pnas.1906817116.

7. Fiorentino M, Sapone A, Senger S, Camhi SS, Kadzielski SM, Buie TM, et al. Blood-brain barrier and intestinal epithelial barrier alterations in autism spectrum disorders. Mol Autism. 2016;7:49. doi: 10.1186/s13229-016-0110-z. PubMed PMID: 27957319; PubMed Central PMCID: PMCPMC5129651.

8. Abdel-Haq R, Schlachetzki JCM, Glass CK, Mazmanian SK. Microbiome-microglia connections via the gut-brain axis. J Exp Med. 2019;216(1):41-59. doi: 10.1084/jem.20180794. PubMed PMID: 30385457; PubMed Central PMCID: PMCPMC6314531.

9. Yano JM, Yu K, Donaldson GP, Shastri GG, Ann P, Ma L, et al. Indigenous bacteria from the gut microbiota regulate host serotonin biosynthesis. Cell. 2015;161(2):264-76. doi: 10.1016/j.cell.2015.02.047. PubMed PMID: 25860609; PubMed Central PMCID: PMCPMC4393509.

10. Jastrzebska-Wiesek M, Partyka A, Rychtyk J, Sniecikowska J, Kolaczkowski M, Wesolowska A, et al. Activity of Serotonin 5-HT1A Receptor Biased Agonists in Rat: Anxiolytic and Antidepressant-like properties. ACS Chem Neurosci. 2018;9(5):1040-50. doi: 10.1021/acschemneuro.7b00443. PubMed PMID: 29266914.

11. Zurita MF, Cardenas PA, Sandoval ME, Pena MC, Fornasini M, Flores N, et al. Analysis of gut microbiome, nutrition and immune status in autism spectrum disorder: a case-control study in Ecuador. Gut Microbes. 2020;11(3):453-64. doi: 10.1080/19490976.2019.1662260. PubMed PMID: 31530087.

12. Adams JB, Johansen LJ, Powell LD, Quig D, Rubin RA. Gastrointestinal flora and gastrointestinal status in children with autism--comparisons to typical children and correlation with autism severity. BMC Gastroenterol. 2011;11:22. doi: 10.1186/1471-230X-11-22. PubMed PMID: 21410934; PubMed Central PMCID: PMCPMC3072352.

13. Kang DW, Ilhan ZE, Isern NG, Hoyt DW, Howsmon DP, Shaffer M, et al. Differences in fecal microbial metabolites and microbiota of children with autism spectrum disorders. Anaerobe. 2018;49:121-31. doi: 10.1016/j.anaerobe.2017.12.007. PubMed PMID: 29274915.

14. Pulikkan J, Maji A, Dhakan DB, Saxena R, Mohan B, Anto MM, et al. Gut Microbial Dysbiosis in Indian Children with Autism Spectrum Disorders. Microb Ecol. 2018;76(4):1102-14. doi: 10.1007/s00248-018-1176-2. PubMed PMID: 29564487.

15. Arnold LE, Luna RA, Williams K, Chan J, Parker RA, Wu Q, et al. Probiotics for Gastrointestinal Symptoms and Quality of Life in Autism: A Placebo-Controlled Pilot Trial. J Child Adolesc Psychopharmacol. 2019;29(9):659-69. doi: 10.1089/cap.2018.0156. PubMed PMID: 31478755; PubMed Central PMCID: PMCPMC7364307.

16. van Wouwe JP, Sanctuary MR, Kain JN, Chen SY, Kalanetra K, Lemay DG, et al. Pilot study of probiotic/colostrum supplementation on gut function in children with autism and gastrointestinal symptoms. PLoS One. 2019;14(1). doi: 10.1371/journal.pone.0210064.

17. Kocsis RN. Book Review: Diagnostic and Statistical Manual of Mental Disorders: Fifth Edition (DSM-5). Int J Offender Ther Comp Criminol. 2013;57(12):1546-8. doi: 10.1177/0306624x13511040.

18. Baron-Cohen S. Early identification of autism by the CHecklist for Autism in Toddlers (CHAT). J R Soc Med. 2000; Oct;93(10):521-5. doi: 10.1177/014107680009301007. PubMed Central PMCID: PMCPMC1298126.

19. Baron-Cohen S, Scott FJ, Allison C, Williams J, Bolton P, Matthews FE, et al. Prevalence of autism-spectrum conditions: UK school-based population study. Br J Psychiatry. 2009;194(6):500-9. doi: 10.1192/bjp.bp.108.059345. PubMed PMID: 19478287.

20. Collaborators GRF. Global, regional, and national comparative risk assessment of 84 behavioural, environmental and occupational, and metabolic risks or clusters of risks for 195 countries and territories, 1990-2017: a systematic analysis for the Global Burden of Disease Study 2017. Lancet. 2018;392(10159):1923-1994. doi: 10.1016/S0140-6736(18)32225-6. PubMed Central PMCID: PMCPMC6227755.

21. Catala-Lopez F, Ridao M, Hurtado I, Nunez-Beltran A, Genova-Maleras R, Alonso-Arroyo A, et al. Prevalence and comorbidity of autism spectrum disorder in Spain: study protocol for a systematic review and meta-analysis of observational studies. Syst Rev. 2019;8(1):141. doi: 10.1186/s13643-019-1061-1. PubMed PMID: 31200773; PubMed Central PMCID: PMCPMC6570970.

22. Baio J WLCD, Maenner MJ, Daniels J, . Prevalence of autism spectrum disorder among children aged 8 years - autism and developmental disabilities monitoring network, 11 sites. MMWR Surveill Summ. 2018;67(6):1-23. doi: 10.15585/mmwr.ss6706a1. PubMed Central PMCID: PMCPMC5919599.

23. Sun X, Allison C, Wei L, Matthews FE, Auyeung B, Wu YY, et al. Autism prevalence in China is comparable to Western prevalence. Mol Autism. 2019;10:7. doi: 10.1186/s13229-018-0246-0. PubMed PMID: 30858963; PubMed Central PMCID: PMCPMC6394100.

24. Buescher AV, Cidav Z, Knapp M, Mandell DS. Costs of autism spectrum disorders in the United Kingdom and the United States. JAMA Pediatr. 2014;168(8):721-8. doi: 10.1001/jamapediatrics.2014.210. PubMed PMID: 24911948.

25. Gibson GR, Hutkins R, Sanders ME, Prescott SL, Reimer RA, Salminen SJ, et al. Expert consensus document: The International Scientific Association for Probiotics and Prebiotics (ISAPP) consensus statement on the definition and scope of prebiotics. Nat Rev Gastroenterol Hepatol. 2017;14(8):491-502. doi: 10.1038/nrgastro.2017.75. PubMed PMID: 28611480.

26. Shaaban SY, El Gendy YG, Mehanna NS, El-Senousy WM, El-Feki HSA, Saad K, et al. The role of probiotics in children with autism spectrum disorder: A prospective, open-label study. Nutr Neurosci. 2018;21(9):676-81. doi: 10.1080/1028415X.2017.1347746. PubMed PMID: 28686541.

27. Liu YW, Liong MT, Chung YE, Huang HY, Peng WS, Cheng YF, et al. Effects of Lactobacillus plantarum PS128 on Children with Autism Spectrum Disorder in Taiwan: A Randomized, Double-Blind, Placebo-Controlled Trial. #N/A. 2019;11(4). Epub 2019/04/14. doi: 10.3390/nu11040820. PubMed PMID: 30979038; PubMed Central PMCID: PMCPMC6521002.

28. Wang Y, Li N, Yang JJ, Zhao DM, Chen B, Zhang GQ, et al. Probiotics and fructo-oligosaccharide intervention modulate the microbiota-gut brain axis to improve autism spectrum reducing also the hyper-serotonergic state and the dopamine metabolism disorder. Pharmacol Res. 2020;157:104784. Epub 2020/04/20. doi: 10.1016/j.phrs.2020.104784. PubMed PMID: 32305492.

29. Marler S, Ferguson BJ, Lee EB, Peters B, Williams KC, McDonnell E, et al. Brief Report: Whole Blood Serotonin Levels and Gastrointestinal Symptoms in Autism Spectrum Disorder. J Autism Dev Disord. 2016;46(3):1124-30. doi: 10.1007/s10803-015-2646-8. PubMed PMID: 26527110; PubMed Central PMCID: PMCPMC4852703.

30. Ballester P, Richdale AL, Baker EK, Peiro AM. Sleep in autism: A biomolecular approach to aetiology and treatment. Sleep Med Rev. 2020;54:101357. doi: 10.1016/j.smrv.2020.101357. PubMed PMID: 32759030.

31. Shaaban SY EGY, Mehanna NS, El-Senousy WM, El-Feki HSA, Saad K, El-Asheer OM. . The role of probiotics in children with autism spectrum disorder: A prospective, open-label study. Nutr Neurosci. 2018; 21(9):676-681. doi: 10.1080/1028415X.2017.1347746.; PubMed Central PMCID: PMC PMID: 28686541.

32. Geier DA, Kern JK, Geier MR. A Comparison of the Autism Treatment Evaluation Checklist (ATEC) and the Childhood Autism Rating Scale (CARS) for the Quantitative Evaluation of Autism. J Ment Health Res Intellect Disabil. 2013;6(4):255-67. doi: 10.1080/19315864.2012.681340. PubMed PMID: 23914277; PubMed Central PMCID: PMCPMC3725669.

33. Moon SJ, Hwang JS, Shin AL, Kim JY, Bae SM, Sheehy-Knight J, et al. Accuracy of the Childhood Autism Rating Scale: a systematic review and meta-analysis. Dev Med Child Neurol. 2019;61(9):1030-8. doi: 10.1111/dmcn.14246. PubMed PMID: 30977125.

34. Owens JA, Spirito A, McGuinn M. The Children's Sleep Habits Questionnaire (CSHQ): psychometric properties of a survey instrument for school-aged children. Sleep. 2015;15;23(8):1043-51. doi: 10.1037/t33022-000. PubMed Central PMCID: PMCPMID: 11145319.

35. Johnson CR, Smith T, DeMand A, Lecavalier L, Evans V, Gurka M, et al. Exploring sleep quality of young children with autism spectrum disorder and disruptive behaviors. Sleep Med. 2018;44:61-6. doi: 10.1016/j.sleep.2018.01.008. PubMed PMID: 29530371; PubMed Central PMCID: PMCPMC5853135.

36. Svedlund J SI, Dotevall G. . GSRS--a clinical rating scale for gastrointestinal symptoms in patients with irritable bowel syndrome and peptic ulcer disease. Dig Dis Sci 1988;33(2):129-134.

37. Lewis SJ, Heaton KW. Stool form scale as a useful guide to intestinal transit time. Scand J Gastroenterol. 1997;32(9):920-4. doi: 10.3109/00365529709011203. PubMed PMID: 9299672.

38. Myers SM, Johnson CP, American Academy of Pediatrics Council on Children With D. Management of children with autism spectrum disorders. Pediatrics. 2007;120(5):1162-82. doi: 10.1542/peds.2007-2362. PubMed PMID: 17967921.

39. Siaperas P HS, Proios P. . Challenging behaviours on people with autism: A case study on the effect of a residential training programme based on structured teaching and TEACCH method. . Psychiatriki. 2007;(4):343-50. PubMed Central PMCID: PMCPMID: 22466678.

40. Yuan J, Song J, Zhu D, Sun E, Xia L, Zhang X, et al. Lithium Treatment Is Safe in Children With Intellectual Disability. Front Mol Neurosci. 2018;11:425. doi: 10.3389/fnmol.2018.00425. PubMed PMID: 30524233; PubMed Central PMCID: PMCPMC6262083.
