# Supplementary material for: The role of probiotics in children with autism spectrum disorders: A study protocol for a randomised controlled trial
Source: PLoS One. 2022 Feb 24;17(2):e0263109. doi: 10.1371/journal.pone.0263109 (PMC8870536; doi:10.1371/journal.pone.0263109)
Supplement: S2 File — (DOCX) [file pone.0263109.s005.docx]

**益生菌在儿童孤独症中的作用及其机制的研究**

**研究方案**

**郑州大学第三附属医院儿科**

**朱长连**

**2019年9月1日**

**益生菌在儿童孤独症中的作用及其机制的研究**

**研究小组**

本项目由河南省小儿脑损伤重点实验室和郑州大学第三附属医院儿童发育行为科合作完成。

**项目负责人**

朱长连 教授，博导，郑州大学第三附属医院儿科，河南 郑州，邮编450052，[电子邮箱zhuc@zzu.edu.cn](mailto:电子邮箱zhuc@zzu.edu.cn)，电话：+86 371 66903051

**主要研究人员**

张玲玲，博士生，河南省小儿脑损伤重点实验室

许毅然，医 师，河南省小儿脑损伤重点实验室

李宏伟，博士生，河南省小儿脑损伤重点实验室

李兵兵，研究员，河南省小儿脑损伤重点实验室

段桂琴，医 师，郑州大学第三附属医院儿童发育行为科

朱长连，医 师，河南省小儿脑损伤重点实验室

**概述**

本研究是一项随机对照研究，主要研究目的是复合益生菌对改善孤独症儿童的核心症状或降低其严重程度的作用。次要研究目的是其对孤独症儿童的伴随症状如胃肠道紊乱、睡眠障碍的调节作用，以及对血液及粪便代谢物、肠道菌群和免疫系统等的影响。

3-12岁符合DSM-5诊断标准的孤独症患儿，排除三个月内服用抗生素及抗真菌药、特殊饮食：如无麸质，酪蛋白饮食，或特殊的碳水化合物饮食、三个月内服用益生元、益生菌或抗氧化剂、腹泻或一周内有发热等明显炎症反应、有其他神经系统症状如癫痫等、克罗恩病，IBD（炎症性肠病），食物不耐受等胃肠道疾病、肝脏疾病、Ⅰ型糖尿病、Rett综合征(RTT)等遗传代谢性疾病，并经家属知情同意后，根据严重程度、性别和年龄分层随机分成治疗组和对照组，治疗组给予三个月的益生菌的治疗及三个月的停药观察，共计六个月；对照组给予等量的麦芽糖糊精，两组患儿疾病的治疗和护理按照相同的诊疗规范。

随访在服用益生菌1月、2月、3月、6月、12月时进行，主要应用ATEC、CSHQ、Birstol等量表来评价孤独症患儿的核心症状、胃肠道及睡眠等情况。主要结果是益生菌能否缓解孤独症患儿的落后的语言和认知、狭窄的兴趣范围及紊乱的胃肠道和睡眠等。

**研究背景**

近年来，肠道菌群引起了人们的广泛关注。它们在肠道内与人保持稳定的共生关系，肠道微生物编码的基因数量是人类细胞基因总数的150倍，被视为“第二大脑”^[1]^。人们发现，肠道菌群通过神经系统^[2]^、免疫系统^[3-6]^和内分泌系统^[7, 8]^，调节认知、社会行为和情感的变化^[9, 10]^。ASD患儿的肠道菌群丰度无论在种间还是种内都被发现发生了很大的变化。研究表明，*Akkermansia*、*Coprococcus* 和 *Ruminococcus*在ASD患儿中丰度升高^[11]^, *Bifidobacterium*水平较低^[12-14]^。通过在饮食中加入*Lactobacillus*和*Bifidobacterium*混合物，自闭症患者的胃肠道症状和生活质量得到了改善^[15, 16]^。

孤独症谱系障碍(autism spectrum disorder，ASD)简称孤独症，与自闭症同义，是一组以社交沟通障碍、兴趣或活动范围狭窄以及重复刻板行为为主要特征的神经发育性障碍^[17]^。ASD的发病率随着时间呈飞速上升的趋势，从2000年的0.31%上升到2009年的1.57%^[18, 19]^。据不完全统计，全球有6220万人患有自闭症^[20]^。并且，在不同的国家和地区表现出不同的发病率;例如，ASD在西班牙的患病率约为1%^[21]^，在美国的患病率约为1.69%^[22]^，在中国的患病率约为1%^[23]^。ASD的核心症状是狭窄的兴趣和刻板的行为，一项纵向研究表明，ASD的预后较差，甚至一些患者不能独立生活，这将严重影响社会交往、学习和工作，无疑会给家庭和社会带来了沉重的负担^[24]^。

益生菌是肠道菌群的重要组成部分，被定义为“活微生物，当给予足够数量时，会给宿主带来健康益处”^[25]^，常见的益生菌：①乳杆菌类（如嗜酸乳杆菌、干酪乳杆菌、詹氏乳杆菌、拉曼乳杆菌等）；②双歧杆菌类（如长双歧杆菌、短双歧杆菌、卵形双歧杆菌、嗜热双歧杆菌等）；③革兰氏阳性球菌（如粪链球菌、乳球菌、中介链球菌等）；④ 一些酵母菌与酶亦可归入益生菌的范畴。近年来，益生菌在ASD患儿中的研究显示，益生菌在改善胃肠功能障碍、营养不良和ASD症状严重程度方面具有巨大的潜在益处^[16, 26-28]^。有趣的是，血液中的5-羟色胺(5-HT)水平与ASD患者胃肠道症状较低相关^[29]^。此外，γ-氨基丁酸（GABA）和褪黑激素促进睡眠，而5-羟色胺、谷氨酸和乙酰胆碱主要负责觉醒^[30]^。相对药物治疗而言，益生菌更加安全^[31]^.

综上所述，肠道菌群可以通过多样性的变化来调节神经递质、激素等肠道代谢物的水平。这可能会对孤独症儿童的行为、兴趣、情绪、胃肠道和睡眠产生影响。然而，具体的调节方式和途径并不清楚。因此，我们推测，补充益生菌可能有助于改善ASD患儿的肠道异常代谢和免疫力，从而改善ASD症状。

本实验用复合益生菌对ASD进行干预，经专业的诊断量表评估患者的临床表征后，进而发现益生菌对ASD患者辅助治疗的价值。然后通过LC-MS非靶标代谢组学、流式细胞技术、宏基因组和宏转录组等多组学方法，从多层面上解析并推测其可能发生的机制，以期为ASD的诊断和治疗提供新的参考。

**创新点**

1. 益生菌类型：由*Lactobacillus*, *Bifidobacterial* 和 *Streptococcus thermophilus*复合而成；
2. 设计亚组分析：包括年龄、性别、严重程度；
3. 主、次症状结合：包括核心症状及胃肠道、睡眠障碍等伴随症状；
4. 观察终点：随访至用药后12个月；
5. 免疫学：包括血清免疫细胞的检测；
6. 代谢组学：包括血清和粪便的代谢产物的测定；
7. 宏基因组学：包括粪便肠道菌群的测定。

**研究目的**

该分层随机对照研究主要有三个研究目的：

1. 研究益生菌对ASD核心症状、胃肠道及睡眠障碍的影响。
2. 研究ASD可能的早期生物学标记物。
3. 研究ASD可能的致病机制。

**研究假设**

主要假设：益生菌可缓解孤独症儿童的核心症状，如：狭窄的兴趣或活动范围和重复刻板的行为等。

次要假设（a）：益生菌可平衡孤独症儿童的胃肠道紊乱，比如便秘，消化不良等；

次要假设（b）：肠道菌群的代谢产物可以改善孤独症儿童的睡眠障碍；

次要假设（c）：肠道菌群的带刺儿产物可以提高孤独症儿童的免疫力；

**研究对象**

入选标准：

1. DSM-5明确诊断为ASD者；
2. 年龄3-12岁，性别不限；
3. 自愿参加本试验并签署知情同意书；

排除标准：

1. 三个月内服用抗生素及抗真菌药；
2. 特殊饮食：如无麸质，酪蛋白饮食，或特殊的碳水化合物饮食；
3. 三个月内服用其它益生元，益生菌，抗氧化剂；
4. 腹泻或一周内有发热等明显炎症反应；
5. 有其他神经系统症状如癫痫等；
6. 克罗恩病，IBD（炎症性肠病），食物不耐受等胃肠道疾病;
7. 肝脏疾病；
8. Ⅰ型糖尿病 ；
9. Rett综合征(RTT)等遗传代谢性疾病的明确诊断;

退出标准

1. 因未按要求服用益生菌或中途服用抗生素的患者；
2. 采集的血样、大便样本非完整生物学信息的患者；
3. 无法完成DSM-5、CARS等量表评估的患者；

满足入选标准且除外排除标准的孤独症患儿根据性别、年龄、严重程度进行随机化分组。

**研究治疗方案**

实验组：口服益生菌混合物50亿CFU/5g，每天两次，早晚各一次，持续3个月。

对照组：等量麦芽糖糊精，疗程和用法同实验组。

两组患儿给予相同的护理和诊疗方案。

**诊疗方法**

诊断标准

符合美国精神病诊断统计手册第5版^[17]^中制定的ASD诊断标准： ①在多种环境下持续表现为社会沟通与社会交往缺陷；②局限、重复的兴趣、行为或活动；③发育早期即存在这些症状；④这些症状引起了职业、社交或其他重要功能方面的显著障碍；⑤智力发育缺陷或整体发育迟缓不能更好地解释这些症状；

满足以上标准可确诊为ASD，其中①②是ASD的核心症状。需经过3位同领域的高年资专家共同诊断患儿为孤独症谱系障碍。

临床疗效评估

使用孤独症疗效评估量表（Autism Treatment Evaluation Checklist， ATEC）^[32]^对ASD患儿临床症状进行评估。该量表包括4个分量表，分别是言语、感知觉、社交、行为，总分0-179分，评分越高则孤独症病情越重。以治疗前后ATEC评分的减分率作为疗效指数（N），N=（治疗前ATEC评分-治疗后ATEC评分）÷治疗前ATEC评分×100%。显效：N≥50%；有效：N： 20%—50%；无效：N<20%。总有效率=（显效+有效）/总例数×100%。

严重程度评估

使用儿童孤独症评定量表（Childhood Autism Rating Scale， CARS）^[33]^。该量表共计60分，分数越高，症状越严重，具体评分标准如下：总分低于30分：初步判断为无孤独症；30-60分：有孤独症；其中30-37分：为轻到中度孤独症；37-60分，并至少有5项的评分高于3分：重度孤独症。

儿童睡眠习惯的评估

本实验将使用儿童睡眠习惯问卷（CSHQ）^[34]^评估患儿睡眠情况，对于有异常睡眠的患儿将进一步通过脑电图进行睡眠检测。该量表从八个不同层面反映儿童常见睡眠问题：就寝习惯、入睡潜伏期、睡眠持续时间、睡眠焦虑、夜醒、异态睡眠、睡眠呼吸障碍、白天嗜睡。问卷采用总分大于41分作为评估睡眠障碍的标准，并在每周超过两个晚上出现条目中的内容才能定义睡眠障碍^[35]^。

胃肠道的评估

本实验将使用胃肠道症状评分问卷（GSRS）^[36]^和Bristol stool chart^[37]^评估ASD患儿的胃肠道的情况。

主要康复训练

康复训练为应用行为分析疗法（ABA）^[38]^和结构化教育（TEACCH）^[39]^。

**样本及数据收集**

血液样本

使用含有肝素抗凝剂的绿色真空采血管收集外周静脉血1-2mL。采集时间为：服用益生菌前一周内、服用益生菌三个月时、停用益生菌三个月时。

大便样本

分别于服用益生菌前，服用益生菌三个月时、停用益生菌三个月时留取粪便样本；1小时内暂存于-20℃冰箱，24小时内冰袋环境下转运，-80℃保存。

尿液样本

分别于服用益生菌前，服用益生菌三个月时、停用益生菌三个月时留取晨尿样本；1小时内暂存于-20℃冰箱，24小时内冰袋环境下转运，-80℃保存。

**数据收集**

符合研究标准的孤独症患儿，需要采集母亲的妊娠史，如妊娠高血压、分娩方式。患儿信息包括性别、年龄、窒息史、家族史、过敏史，DSM-5诊断结果及ATEC、CARS、CSHQ、GSRS、PSI-SF的评估结果。这些信息可用来进行亚组分析。

**随访**

1，2,3,6,12月：胃肠道、睡眠、语言表达和沟通能力、社交能力、感知能力、健康和生理行为等的评估（量表法）。

**主要评估指标**

1. 疗效评估
2. 疾病的严重程度

**次要评估指标**

1. 胃肠道症状
2. 睡眠
3. 父母养育压力
4. 饮食习惯
5. 饮食结构
6. 血液及粪便代谢产物
7. 免疫细胞
8. 肠道菌群丰度

**伦理问题**

研究方案及有关资料经过郑州大学第三附属医院伦理委员会批准（2020-56），受试者进入研究前，研究者向患儿监护人说明本项临床研究的详细情况，并签署知情同意书。

**随机化**

随机分组根据严重程度、性别、年龄(3-6岁或7-12岁)进行分层。参与者将被分配(1:1)到益生菌组或对照组，在每一个层级将实行独立的随机分组，随机数表由Python(一种跨平台的计算机编程语言)产生。安慰剂组除了成分不一样外，包装、味道和重量都与实验组相同。所有参与者和医生都将对治疗的分配组均不知情。

**样本量**

将使用epitool计算样本大小(https://epitools.ausvet.com.au/onemean)。益生菌组和对照组之间的标准差是基于之前的研究，其中ASD组行为评分的标准偏差值为8.32 ^[31]^。本研究选择α值为0.05，双侧检验，β值为0.1，根据样本量计算公式得每组需67名儿童。根据临床研究经验^[40]^，我们假设因感染、疗效、患者的依从性、抗生素使用或其他不可预见因素导致的脱落病例率为19%，这意味着每组至少有80名儿童，即估计的总样本量为160例。

**实施和监测**

本试验的研究人员由儿童康复专业的高年资主治医师或副主任医师组成，能熟练掌握孤独症的管理和诊疗方案，并能对孤独症患儿的相关并发症做出正确的诊断。专科检查人员由相应科室的1-2名主治医师以上职称的医师完成，并对所有的报告进行校正。数据的收集和监测均由小儿脑损伤重点实验室相关负责人负责。中期分析和安全性数据将由安全管理委员会审查。

**统计学分析**

采用SPSS19.0统计学软件进行数据分析。计量资料采用均数±标准差表示，比较采用双侧t检验；相关性分析采用Pearson相关性检验。计数资料的比较采用卡方检验。以上检验均为双侧检验，P＜0.05为具有统计学差异。

宏基因组测序数据将使用meta-seq宏基因组测序管道进行分析。GTDB-tk(版本1.0.2)将用于识别获得的宏基因组组装基因组的谱系分类。原始UPLC-Q-TOF/MS数据将使用ProteoWizard msConverte工具转换为mzXML文件，然后使用XCMS在线软件进行处理。将每个代谢物进行单位方差标化后，通过Multi Experiment Viewer软件4.9.0得到代谢物的热图。在线KEGG数据库(http://www.genome.jp/kegg/)将用于代谢途径的鉴定。

**参考文献**

1. Qin J, Li R, Raes J, Arumugam M, Burgdorf KS, Manichanh C, et al. A human gut microbial gene catalogue established by metagenomic sequencing. Nature. 2010;464(7285):59-65. doi: 10.1038/nature08821. PubMed PMID: 20203603; PubMed Central PMCID: PMCPMC3779803.

2. Han W, Tellez LA, Perkins MH, Perez IO, Qu T, Ferreira J, et al. A Neural Circuit for Gut-Induced Reward. Cell. 2018;175(3):665-78 e23. doi: 10.1016/j.cell.2018.08.049. PubMed PMID: 30245012; PubMed Central PMCID: PMCPMC6195474.

3. Mukherji A, Kobiita A, Ye T, Chambon P. Homeostasis in intestinal epithelium is orchestrated by the circadian clock and microbiota cues transduced by TLRs. Cell. 2013;153(4):812-27. doi: 10.1016/j.cell.2013.04.020. PubMed PMID: 23663780.

4. Erny D, Hrabe de Angelis AL, Jaitin D, Wieghofer P, Staszewski O, David E, et al. Host microbiota constantly control maturation and function of microglia in the CNS. Nat Neurosci. 2015;18(7):965-77. doi: 10.1038/nn.4030. PubMed PMID: 26030851; PubMed Central PMCID: PMCPMC5528863.

5. Lamas B, Richard ML, Leducq V, Pham HP, Michel ML, Da Costa G, et al. CARD9 impacts colitis by altering gut microbiota metabolism of tryptophan into aryl hydrocarbon receptor ligands. Nat Med. 2016;22(6):598-605. doi: 10.1038/nm.4102. PubMed PMID: 27158904; PubMed Central PMCID: PMCPMC5087285.

6. Irene Tsilionia ABP, b, Harry Pantazopoulosc,1, Sabina Berrettac, Pio Contid, Susan E. Leemane,2,, and Theoharis C. Theoharidesa b, f,2. IL-37 is increased in brains of children with autism spectrum disorder and inhibits human microglia stimulated by neurotensin. PNAS. 2019. doi: 10.1073/pnas.1906817116.

7. Fiorentino M, Sapone A, Senger S, Camhi SS, Kadzielski SM, Buie TM, et al. Blood-brain barrier and intestinal epithelial barrier alterations in autism spectrum disorders. Mol Autism. 2016;7:49. doi: 10.1186/s13229-016-0110-z. PubMed PMID: 27957319; PubMed Central PMCID: PMCPMC5129651.

8. Abdel-Haq R, Schlachetzki JCM, Glass CK, Mazmanian SK. Microbiome-microglia connections via the gut-brain axis. J Exp Med. 2019;216(1):41-59. doi: 10.1084/jem.20180794. PubMed PMID: 30385457; PubMed Central PMCID: PMCPMC6314531.

9. Yano JM, Yu K, Donaldson GP, Shastri GG, Ann P, Ma L, et al. Indigenous bacteria from the gut microbiota regulate host serotonin biosynthesis. Cell. 2015;161(2):264-76. doi: 10.1016/j.cell.2015.02.047. PubMed PMID: 25860609; PubMed Central PMCID: PMCPMC4393509.

10. Jastrzebska-Wiesek M, Partyka A, Rychtyk J, Sniecikowska J, Kolaczkowski M, Wesolowska A, et al. Activity of Serotonin 5-HT1A Receptor Biased Agonists in Rat: Anxiolytic and Antidepressant-like properties. ACS Chem Neurosci. 2018;9(5):1040-50. doi: 10.1021/acschemneuro.7b00443. PubMed PMID: 29266914.

11. Zurita MF, Cardenas PA, Sandoval ME, Pena MC, Fornasini M, Flores N, et al. Analysis of gut microbiome, nutrition and immune status in autism spectrum disorder: a case-control study in Ecuador. Gut Microbes. 2020;11(3):453-64. doi: 10.1080/19490976.2019.1662260. PubMed PMID: 31530087.

12. Adams JB, Johansen LJ, Powell LD, Quig D, Rubin RA. Gastrointestinal flora and gastrointestinal status in children with autism--comparisons to typical children and correlation with autism severity. BMC Gastroenterol. 2011;11:22. doi: 10.1186/1471-230X-11-22. PubMed PMID: 21410934; PubMed Central PMCID: PMCPMC3072352.

13. Kang DW, Ilhan ZE, Isern NG, Hoyt DW, Howsmon DP, Shaffer M, et al. Differences in fecal microbial metabolites and microbiota of children with autism spectrum disorders. Anaerobe. 2018;49:121-31. doi: 10.1016/j.anaerobe.2017.12.007. PubMed PMID: 29274915.

14. Pulikkan J, Maji A, Dhakan DB, Saxena R, Mohan B, Anto MM, et al. Gut Microbial Dysbiosis in Indian Children with Autism Spectrum Disorders. Microb Ecol. 2018;76(4):1102-14. doi: 10.1007/s00248-018-1176-2. PubMed PMID: 29564487.

15. Arnold LE, Luna RA, Williams K, Chan J, Parker RA, Wu Q, et al. Probiotics for Gastrointestinal Symptoms and Quality of Life in Autism: A Placebo-Controlled Pilot Trial. J Child Adolesc Psychopharmacol. 2019;29(9):659-69. doi: 10.1089/cap.2018.0156. PubMed PMID: 31478755; PubMed Central PMCID: PMCPMC7364307.

16. van Wouwe JP, Sanctuary MR, Kain JN, Chen SY, Kalanetra K, Lemay DG, et al. Pilot study of probiotic/colostrum supplementation on gut function in children with autism and gastrointestinal symptoms. PLoS One. 2019;14(1). doi: 10.1371/journal.pone.0210064.

17. Kocsis RN. Book Review: Diagnostic and Statistical Manual of Mental Disorders: Fifth Edition (DSM-5). Int J Offender Ther Comp Criminol. 2013;57(12):1546-8. doi: 10.1177/0306624x13511040.

18. Baron-Cohen S. Early identification of autism by the CHecklist for Autism in Toddlers (CHAT). J R Soc Med. 2000; Oct;93(10):521-5. doi: 10.1177/014107680009301007. PubMed Central PMCID: PMCPMC1298126.

19. Baron-Cohen S, Scott FJ, Allison C, Williams J, Bolton P, Matthews FE, et al. Prevalence of autism-spectrum conditions: UK school-based population study. Br J Psychiatry. 2009;194(6):500-9. doi: 10.1192/bjp.bp.108.059345. PubMed PMID: 19478287.

20. Collaborators GRF. Global, regional, and national comparative risk assessment of 84 behavioural, environmental and occupational, and metabolic risks or clusters of risks for 195 countries and territories, 1990-2017: a systematic analysis for the Global Burden of Disease Study 2017. Lancet. 2018;392(10159):1923-1994. doi: 10.1016/S0140-6736(18)32225-6. PubMed Central PMCID: PMCPMC6227755.

21. Catala-Lopez F, Ridao M, Hurtado I, Nunez-Beltran A, Genova-Maleras R, Alonso-Arroyo A, et al. Prevalence and comorbidity of autism spectrum disorder in Spain: study protocol for a systematic review and meta-analysis of observational studies. Syst Rev. 2019;8(1):141. doi: 10.1186/s13643-019-1061-1. PubMed PMID: 31200773; PubMed Central PMCID: PMCPMC6570970.

22. Baio J WLCD, Maenner MJ, Daniels J, . Prevalence of autism spectrum disorder among children aged 8 years - autism and developmental disabilities monitoring network, 11 sites. MMWR Surveill Summ. 2018;67(6):1-23. doi: 10.15585/mmwr.ss6706a1. PubMed Central PMCID: PMCPMC5919599.

23. Sun X, Allison C, Wei L, Matthews FE, Auyeung B, Wu YY, et al. Autism prevalence in China is comparable to Western prevalence. Mol Autism. 2019;10:7. doi: 10.1186/s13229-018-0246-0. PubMed PMID: 30858963; PubMed Central PMCID: PMCPMC6394100.

24. Buescher AV, Cidav Z, Knapp M, Mandell DS. Costs of autism spectrum disorders in the United Kingdom and the United States. JAMA Pediatr. 2014;168(8):721-8. doi: 10.1001/jamapediatrics.2014.210. PubMed PMID: 24911948.

25. Reid G, Gadir AA, Dhir R. Probiotics: Reiterating What They Are and What They Are Not. Front Microbiol. 2019;10:424. doi: 10.3389/fmicb.2019.00424. PubMed PMID: 30930863; PubMed Central PMCID: PMCPMC6425910.

26. Shaaban SY, El Gendy YG, Mehanna NS, El-Senousy WM, El-Feki HSA, Saad K, et al. The role of probiotics in children with autism spectrum disorder: A prospective, open-label study. Nutr Neurosci. 2018;21(9):676-81. doi: 10.1080/1028415X.2017.1347746. PubMed PMID: 28686541.

27. Liu YW, Liong MT, Chung YE, Huang HY, Peng WS, Cheng YF, et al. Effects of Lactobacillus plantarum PS128 on Children with Autism Spectrum Disorder in Taiwan: A Randomized, Double-Blind, Placebo-Controlled Trial. #N/A. 2019;11(4). Epub 2019/04/14. doi: 10.3390/nu11040820. PubMed PMID: 30979038; PubMed Central PMCID: PMCPMC6521002.

28. Wang Y, Li N, Yang JJ, Zhao DM, Chen B, Zhang GQ, et al. Probiotics and fructo-oligosaccharide intervention modulate the microbiota-gut brain axis to improve autism spectrum reducing also the hyper-serotonergic state and the dopamine metabolism disorder. Pharmacol Res. 2020;157:104784. Epub 2020/04/20. doi: 10.1016/j.phrs.2020.104784. PubMed PMID: 32305492.

29. Marler S, Ferguson BJ, Lee EB, Peters B, Williams KC, McDonnell E, et al. Brief Report: Whole Blood Serotonin Levels and Gastrointestinal Symptoms in Autism Spectrum Disorder. J Autism Dev Disord. 2016;46(3):1124-30. doi: 10.1007/s10803-015-2646-8. PubMed PMID: 26527110; PubMed Central PMCID: PMCPMC4852703.

30. Ballester P, Richdale AL, Baker EK, Peiro AM. Sleep in autism: A biomolecular approach to aetiology and treatment. Sleep Med Rev. 2020;54:101357. doi: 10.1016/j.smrv.2020.101357. PubMed PMID: 32759030.

31. Shaaban SY EGY, Mehanna NS, El-Senousy WM, El-Feki HSA, Saad K, El-Asheer OM. . The role of probiotics in children with autism spectrum disorder: A prospective, open-label study. Nutr Neurosci. 2018; 21(9):676-681. doi: 10.1080/1028415X.2017.1347746.; PubMed Central PMCID: PMC PMID: 28686541.

32. Geier DA, Kern JK, Geier MR. A Comparison of the Autism Treatment Evaluation Checklist (ATEC) and the Childhood Autism Rating Scale (CARS) for the Quantitative Evaluation of Autism. J Ment Health Res Intellect Disabil. 2013;6(4):255-67. doi: 10.1080/19315864.2012.681340. PubMed PMID: 23914277; PubMed Central PMCID: PMCPMC3725669.

33. Moon SJ, Hwang JS, Shin AL, Kim JY, Bae SM, Sheehy-Knight J, et al. Accuracy of the Childhood Autism Rating Scale: a systematic review and meta-analysis. Dev Med Child Neurol. 2019;61(9):1030-8. doi: 10.1111/dmcn.14246. PubMed PMID: 30977125.

34. Owens JA, Spirito A, McGuinn M. The Children's Sleep Habits Questionnaire (CSHQ): psychometric properties of a survey instrument for school-aged children. Sleep. 2015;15;23(8):1043-51. doi: 10.1037/t33022-000. PubMed Central PMCID: PMCPMID: 11145319.

35. Johnson CR, Smith T, DeMand A, Lecavalier L, Evans V, Gurka M, et al. Exploring sleep quality of young children with autism spectrum disorder and disruptive behaviors. Sleep Med. 2018;44:61-6. doi: 10.1016/j.sleep.2018.01.008. PubMed PMID: 29530371; PubMed Central PMCID: PMCPMC5853135.

36. Svedlund J SI, Dotevall G. . GSRS--a clinical rating scale for gastrointestinal symptoms in patients with irritable bowel syndrome and peptic ulcer disease. Dig Dis Sci 1988;33(2):129-134.

37. Lewis SJ, Heaton KW. Stool form scale as a useful guide to intestinal transit time. Scand J Gastroenterol. 1997;32(9):920-4. doi: 10.3109/00365529709011203. PubMed PMID: 9299672.

38. Myers SM, Johnson CP, American Academy of Pediatrics Council on Children With D. Management of children with autism spectrum disorders. Pediatrics. 2007;120(5):1162-82. doi: 10.1542/peds.2007-2362. PubMed PMID: 17967921.

39. Siaperas P HS, Proios P. . Challenging behaviours on people with autism: A case study on the effect of a residential training programme based on structured teaching and TEACCH method. . Psychiatriki. 2007;(4):343-50. PubMed Central PMCID: PMCPMID: 22466678.

40. Yuan J, Song J, Zhu D, Sun E, Xia L, Zhang X, et al. Lithium Treatment Is Safe in Children With Intellectual Disability. Front Mol Neurosci. 2018;11:425. doi: 10.3389/fnmol.2018.00425. PubMed PMID: 30524233; PubMed Central PMCID: PMCPMC6262083.
